# High-resolution strain rate mapping around inland plate boundary within a volcanic arc using L-band InSAR and dense GNSS networks

**DOI:** 10.1038/s41598-026-48775-x

**Published:** 2026-05-07

**Authors:** Shogo Nagaoka, Youichiro Takada, Takuya Nishimura, Takeshi Sagiya, Yusaku Ohta

**Affiliations:** 1 Department of Natural History Sciences, Hokkaido University, N10W8, Kita-ku, Sapporo, 060-0810 Japan; 2 Department of Earth and Planetary Sciences, Hokkaido University, N10W8, Kita-ku, Sapporo, 060-0810 Japan; 3 Disaster Prevention Research Institute, Kyoto University, Gokasho, Uji, Kyoto 611-0011 Japan; 4 Disaster Mitigation Research Center, Nagoya University, Furocho, Chikusa-ku, Nagoya, 464-8601 Japan; 5 Research Center for Prediction of Earthquakes and Volcanic Eruptions, Graduate School of Science, Tohoku University, 6-6 Aza-Aoba, Aramaki, Aoba-ku, Sendai, 980-8578 Japan

**Keywords:** Interseismic strain rate, ALOS-2 InSAR, Dense GNSS observation, Itoigawa–Shizuoka tectonic line, Niigata–Kobe tectonic zone, Volcanic arc deformation, Natural hazards, Solid Earth sciences

## Abstract

Crustal deformation in central Japan occurs in a highly complex manner characterized by the Itoigawa–Shizuoka Tectonic Line (ISTL), the geologic boundary between the North American and the Eurasian plates. In addition, a prominent strain concentration zone, known as the Niigata–Kobe tectonic zone (NKTZ), has been identified by GNSS surveys. The existence of a volcanic chain introduces another complexity through its rheological heterogeneity. In this region, the spatial resolution and accuracy of strain rate mapping by conventional ground-based geodetic surveys have been limited by dense vegetation and rugged topography. Here, we integrate L-band InSAR data (2014–2023), which can resolve deformation in densely vegetated mountainous areas where ground-based measurements alone are spatially sparse, with GNSS data from a dense network (average spacing of $\sim 10~\textrm{km}$) including private-sector stations, to generate a high-resolution interseismic strain rate map. We identify sharply localized strain rates along the northern ISTL ($\sim 5\times 10^{-7}~\mathrm {yr^{-1}}$) and parts of the NKTZ ($\sim 4\times 10^{-7}~\mathrm {yr^{-1}}$), which are of larger magnitude and more spatially confined than GNSS-only estimates. We also find a distinct strain rate concentration ($\sim 3\times 10^{-7}~\mathrm {yr^{-1}}$) along the southern extension of the Hida Mountains volcanic zone. Some of these concentrated strain rate zones align with known active faults, while others do not (e.g., the southern extension of the Hida range). This study presents the first L-band InSAR-based strain rate map in a densely vegetated mountainous region, revealing the complex deformation style in volcanic arcs and providing new insights into strain accumulation processes.

## Introduction

**Fig. 1:**
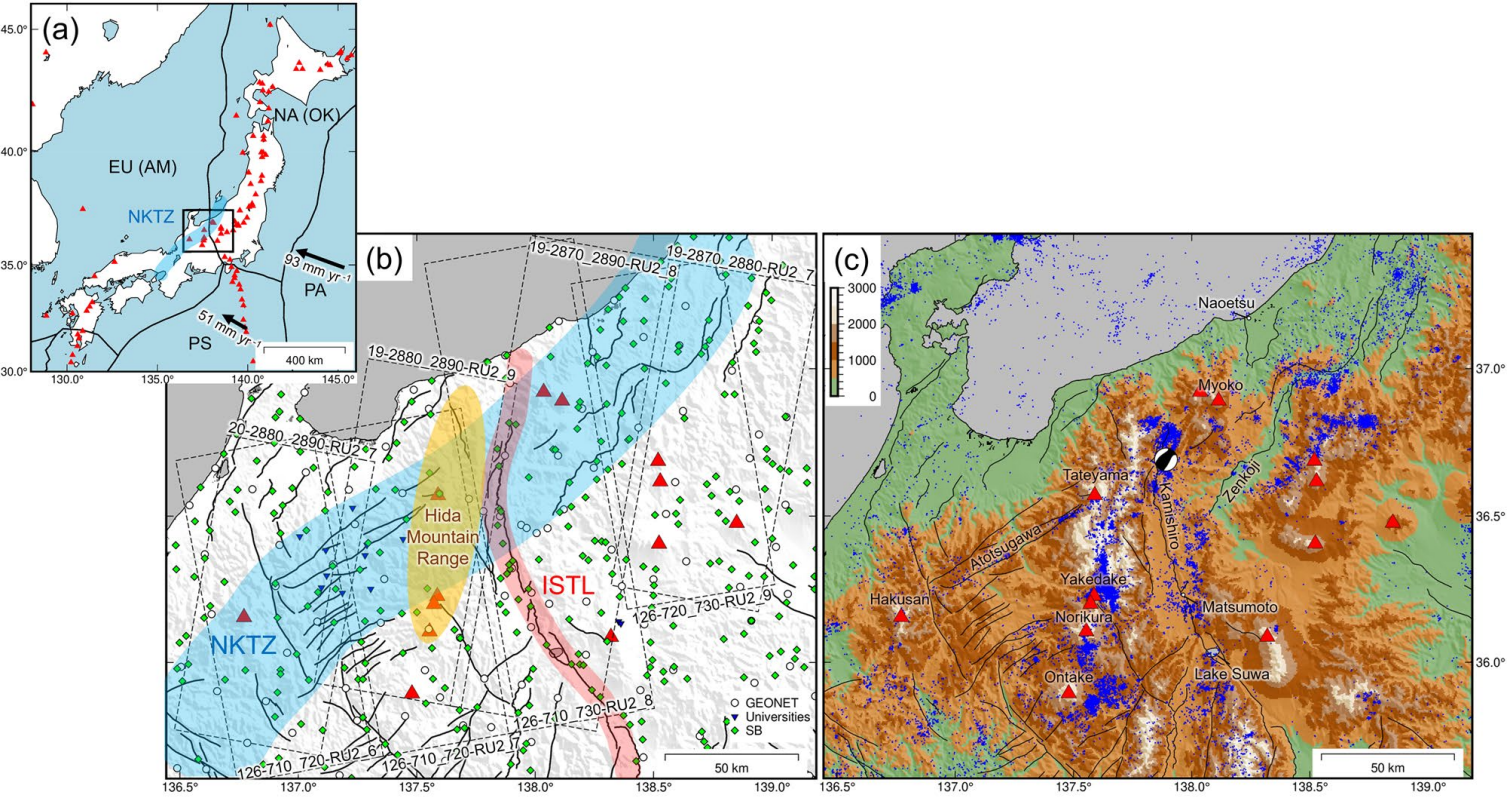
Tectonic settings of central Japan. (**a**) Plate boundaries are shown by black lines^12^. The solid black rectangle marks the area of interest. Black arrows show each plate motion relative to the Eurasian plate^13^. (**b**) Tectonic features and observation coverage. Dashed rectangles denote SAR acquisition frames (SM1 mode; Supplementary Table S1). White, blue and green markers indicate GNSS stations from GEONET, Universities (Hokkaido University, Kyoto University, and Nagoya University), and the private sector (SoftBank Corp.: referred to as SB) networks, respectively. (**c**) Topography of the study area. Blue dots denote epicenters from the JMA unified hypocenter catalog with $M\ge 1$ and $\textrm{depth}\le 20~\textrm{km}$ during 2014–2023. Black lines in (**b**) and (**c**) denote active fault traces^14^ and red triangles indicate Holocene volcanoes^15^. The black focal mechanism represents the 2014 Northern Nagano earthquake ($M_w$ 6.2)^16^. EU (AM), Eurasian (Amur) plate; NA (OK), North American (Okhotsk) plate; PA, Pacific plate; PS, Philippine Sea plate. NKTZ: Niigata–Kobe Tectonic Zone, ISTL: Itoigawa–Shizuoka Tectonic Line. Maps in this figure were generated with the Generic Mapping Tools version 6 (https://www.generic-mapping-tools.org/, accessed on 6 April 2026).

Continental crust is often deformed in a block-like manner, segmented by large active faults^1,2^. However, in many cases, deformation occurs in a more distributed and complex fashion^3–7^. In such cases, understanding where and how strain rates are distributed is critically important for seismic hazard assessments as well as understanding landscape evolution mechanisms. As described below, central Japan serves as a representative example of such complex deformation (Fig. 1). This study aims to quantitatively clarify the style of strain rate accumulation around the inland plate boundary in central Japan.

In central Japan, the geological boundary between the North American plate and the Eurasian plate is known as the Itoigawa–Shizuoka Tectonic Line (ISTL; Fig. 1). The ISTL is characterized by a north–south trending zone of active faults and fold belts^8^. West of the ISTL lies the Hida mountain range, with elevations reaching 2,000 to 3,000 meters. Due to the rugged topography and dense vegetation, the present-day deformation field in this area remains largely unresolved. Strain accumulation across the ISTL has been estimated using geodetic triangulation (1902–1989), with particularly high strain reported near active faults in the northern segment^8–10^. However, these measurements are mostly limited within a narrow valley along the ISTL, making it impossible to constrain the width of the strain concentration zone. GNSS data have also revealed strain rate concentration in the northern segment, but there are few observation points within the Hida mountain range as well^11^. Due to the wide spacing of observation points in both triangulation and GNSS datasets, it remains unclear to what extent strain rate is localized around active faults and folds^11^, or alternatively reflects more broadly distributed deformation accommodated by smaller-scale structures.

Another major strain rate concentration zone in central Japan is the Niigata–Kobe Tectonic Zone (NKTZ), identified from GNSS data^17^ (Fig. 1), which runs in an ENE–WSW direction and intersects the northern part of the ISTL. The NKTZ also lies within mountainous terrain, making ground-based observations difficult. As a result, geological structures responsible for the strain rate concentration are still unclear. In particular, if the deformation is accommodated by numerous short faults and folds, no single dominant structure may be identifiable^4^. For example, the NKTZ crosses the Hida mountain range where no active faults running parallel to the NKTZ have been identified.

To evaluate the role of geological structures in crustal deformation, it is essential to resolve their spatial relationship with the strain rate field over a broad area and at high spatial resolution. At the same time, detailed mapping of the strain rate field can also help determine whether deformation is governed by discrete faults or instead by distributed processes. In recent years, high-resolution strain rate mapping combining InSAR and GNSS has been intensively applied to large-scale continental deformation zones in Turkey^18,19^, Tibet^20–22^, and northern Andes^23,24^. However, these regions are all arid or semi-arid. In densely vegetated and mountainous areas like Japan, it remains extremely challenging to estimate the strain rates using InSAR, and such efforts have only been realized in Taiwan^25^.

In this study, we combine L-band InSAR, whose radio waves can penetrate vegetation, with GNSS observations to investigate the spatial distribution of crustal strain rate near the inland plate boundary in central Japan at high spatial resolution and wide areal coverage. By incorporating a dense GNSS network operated by a private company, we further enhance the spatial resolution. This study also represents, to the best of our knowledge, the first attempt to use L-band InSAR to estimate the strain rates across a volcanic arc. We further discuss rheological heterogeneities of the volcanic arc on the strain rate distribution.

## Material and methods

### SAR data processing

We used high-resolution SAR data (SM1 mode) acquired by the Advanced Land Observing Satellite 2 (ALOS-2) from 2014 to 2023 (Fig. 1b; Supplementary Table S1). Interferograms were produced using the GAMMA software^26^. Image coregistration and topographic phase removal were performed using a 10-meter digital elevation model (DEM) provided by the Geospatial Information Authority of Japan (GSI). To mitigate ionospheric phase noise, which is particularly prominent in L-band SAR data, we applied the split-spectrum method^27^, following an improved procedure^28^ with more robust outlier removal and filtering. Tropospheric phase delays were corrected using the Generic Atmospheric Correction Online Service (GACOS) products^29^.

A time series analysis^30^ was applied to the corrected interferograms to further suppress residual random noise in the line-of-sight (LOS) displacements (Supplementary Text S1). Interferometric pairs were used except for scenes manually identified as being severely affected by residual noise (Supplementary Fig. S1). For each frame, we set the reference point in a flat area, where a GNSS station locates and the interferogram coherence is relatively high. By fitting a linear line to the time series (Supplementary Fig. S2), we suppressed the remaining noise and obtained averaged, long-term interseismic LOS velocities.

Following these procedures, the LOS velocities were independently derived for each satellite path. Since the reference points in the InSAR time series differ among tracks, discontinuities arise between the adjacent velocity maps. To correct these inter-frame gaps, we estimated constant offsets from velocity differences within the overlapping areas and mosaicked the LOS velocities into a common reference frame. After concatenation, long-wavelength residual noise were further corrected using GNSS data, and the resulting velocities were tied into the GNSS reference frame (Supplementary Text S2, Supplementary Fig. S3).

### GNSS data processing

**Fig. 2:**
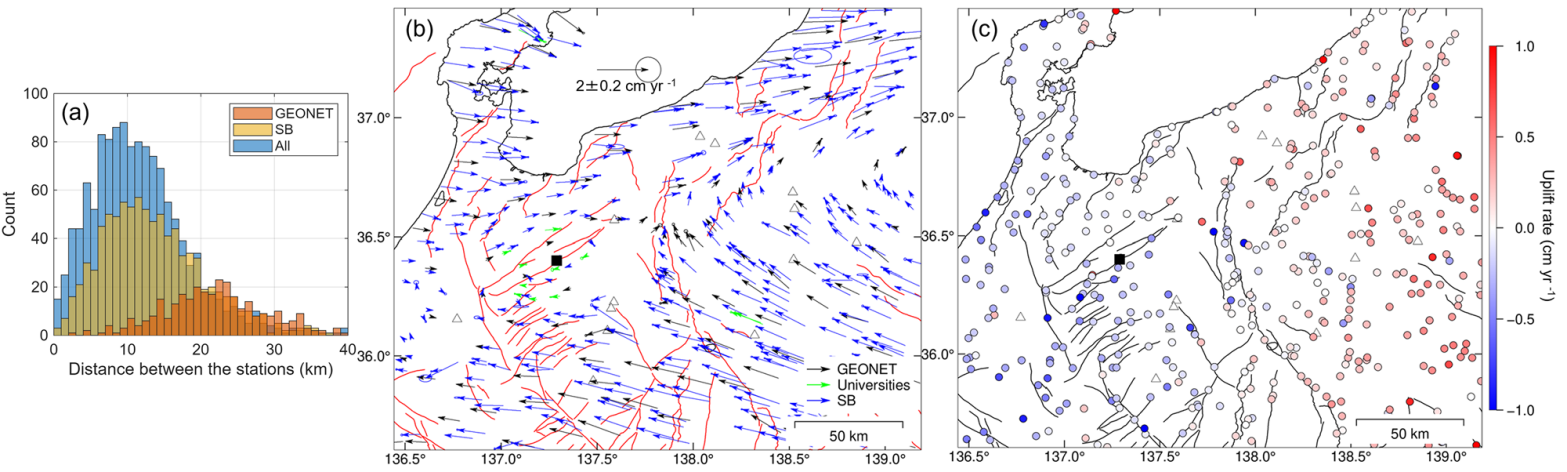
Station distribution and velocities of the GNSS observations. (**a**) Spatial density of the GNSS networks. Neighboring station distances are calculated using Delaunay triangulation. (**b**) Horizontal GNSS velocities relative to a fixed station in the area (black square in the map). The error ellipses are plotted in 95% confidence intervals. (**c**) Vertical GNSS velocities relative to the station (black square). Red (**b**) and black (**c**) lines indicate active fault traces and white triangles indicate Holocene volcanoes. Maps in this figure were generated using the generic mapping tools version 6 (https://www.generic-mapping-tools.org/, accessed on 6 April 2026).

We used GNSS data from three continuous observation networks (Fig. 1b): (1) GNSS Earth Observation Network System (GEONET) operated by the GSI, (2) stations operated by the authors (Hokkaido University, Kyoto University, and Nagoya University), and (3) stations operated by a private-sector company^31,32^ (SoftBank Corp.; hereafter referred to as SB). As shown in Fig. 2a, while the average distance for the GEONET stations is approximately 20–25 km, integrating all three networks results in an unprecedentedly dense network with an average spacing of $\sim 10~\textrm{km}$.

The daily coordinates of all GNSS stations were estimated in the ITRF2014 reference frame using the precise point positioning with ambiguity resolution (PPP-AR) method implemented in the GipsyX software package^33^. Reference GPS satellite orbit and clock information were obtained from the final precise products provided by the Jet Propulsion Laboratory (JPL). Details of the processing parameters and settings are summarized in Supplementary Table S2. Recent evaluation of the same PPP-AR strategy for dense GNSS networks in Japan showed horizontal velocity uncertainties of $\le 0.1~\mathrm {mm~yr^{-1}}$ from $\sim 4\text {-year}$ time series, supporting its applicability to tectonic deformation studies^34^. To estimate interseismic velocities, time series of the daily coordinates were modeled by the following function (Supplementary Fig. S4):

1 $$\begin{aligned} z = c_1 + c_2 t + c_3\sin (2\pi t)+c_4\cos (2\pi t) + c_5\sin (4\pi t) + c_6\cos (4\pi t), \end{aligned}$$

where *z* represents the daily coordinates, *t* is the elapsed time since the first observation (years), and $c_1, c_2,..., c_6$ are the model parameters. We used daily coordinates from 2021 to 2023 for the GEONET and the University networks. The analysis period was limited to data up to 31 December 2023 in order to avoid contamination from the coseismic signal of the 2024 Noto Peninsula earthquake ($M_w$ 7.5), which occurred on 1 January 2024. For the SB network, the daily coordinates are available after 2 May 2021. We compared neighboring stations from different networks (Supplementary Fig. S4), and confirmed that the time series from the GEONET and the SB networks are highly consistent. It is well known that GNSS time series after 2011 are influenced by the postseismic deformation following the 2011 Tohoku-Oki earthquake^35,36^. The time series from 2021 to 2023, however, exhibit minimal temporal variations and are generally well approximated by linear trends (Supplementary Fig. S4), indicating minimal postseismic deformation following the Tohoku-Oki earthquake. In this study, we used the linear term $c_2$ as an average velocity, since the postseismic deformation is spatially smooth in a distant area^37,38^ and has a limited impact on the horizontal strain rates. The GNSS velocity field was first estimated in the ITRF2014 reference frame and then expressed relative to station MAKI, a university station in the study area (black square in Fig. 2b, c), which was used as the reference (fixed) station to reduce common-mode errors. The resultant velocity fields are shown in Fig. 2b,c.

**Fig. 3:**
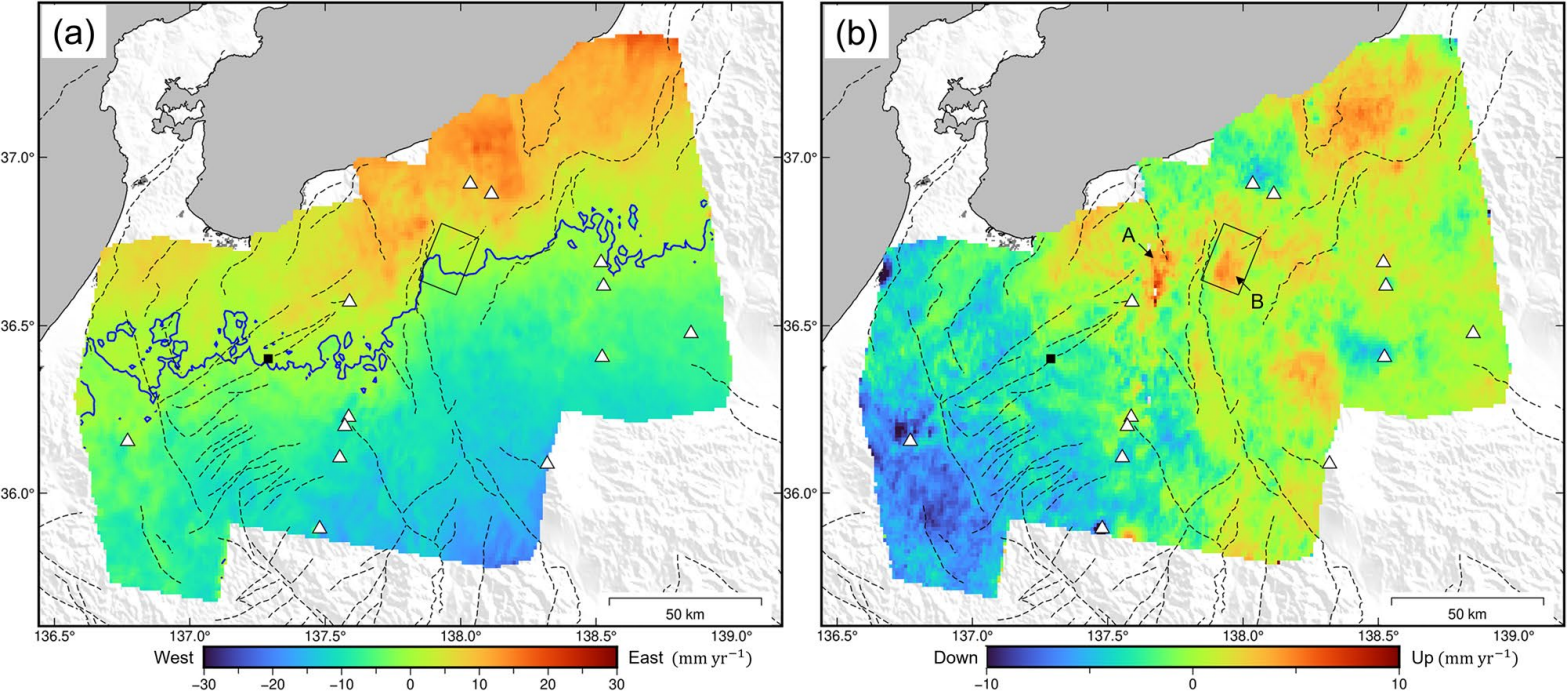
Interseismic velocity field derived from InSAR. (**a**) Eastward velocity. The blue contour line indicates $0~\mathrm {mm~yr^{-1}}$. (**b**) Uplift rate. Note that the color scale differs between panels (**a**) and (**b**). The black square marks the reference station (MAKI), to which all displayed velocities are tied. The white triangles indicate Holocene volcanoes. The dotted lines indicate active fault traces. The black arrows with ”A” and ”B” point to localized uplift north of the Hida mountain range and on the eastern side of the ISTL, respectively. The black rectangle shows the location of the fault model of the 2014 Northern Nagano earthquake^39^. Maps in this figure were generated using the Generic Mapping Tools version 6 (https://www.generic-mapping-tools.org/, accessed on 6 April 2026).

### Horizontal strain rate estimation

We used the GNSS data to further remove spatially long-wavelength residual noise from the InSAR velocities^40,41^ (Supplementary Text S2). The corrected ascending and descending LOS velocities were then decomposed into eastward and vertical velocities^42^ (Fig. 3).

**Fig. 4:**
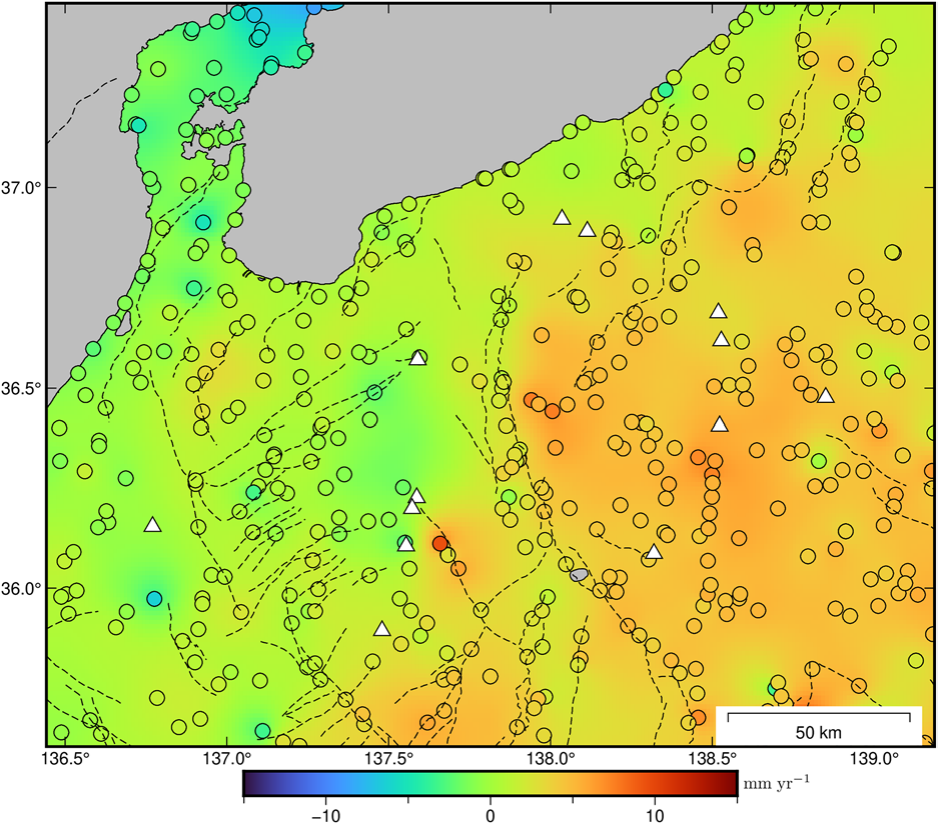
Interpolated GNSS northward velocities. The white triangles indicate Holocene volcanoes. The dotted lines indicate active fault traces. Map in this figure was generated using the Generic Mapping Tools version 6 (https://www.generic-mapping-tools.org/, accessed on 6 April 2026).

Subsequently, we estimated strain rate using the eastward velocity from InSAR and the northward velocity from GNSS^21,25^. First, we defined a regular grid with 1 km spacing across the study region. The eastward InSAR velocities were then subsampled onto this grid (Fig. 3a). Next, the GNSS northward velocities (Fig. 2b) were interpolated onto the same grid using the Kriging method^43^ (Fig. 4; Supplementary Text S3). To derive strain rates from these discrete velocity measurements, it is necessary to calculate spatial gradients. Direct finite differencing of neighboring grid points strongly amplifies short-wavelength noise, so we instead adopted a local plane-fitting approach (Supplementary Text S4). For each grid point, we selected data within a circular area of radius *R* and estimated spatial gradients by fitting the following function using a least-squares method:

2 $$\begin{aligned} z = ax + by + c \end{aligned}$$

**Fig. 5:**
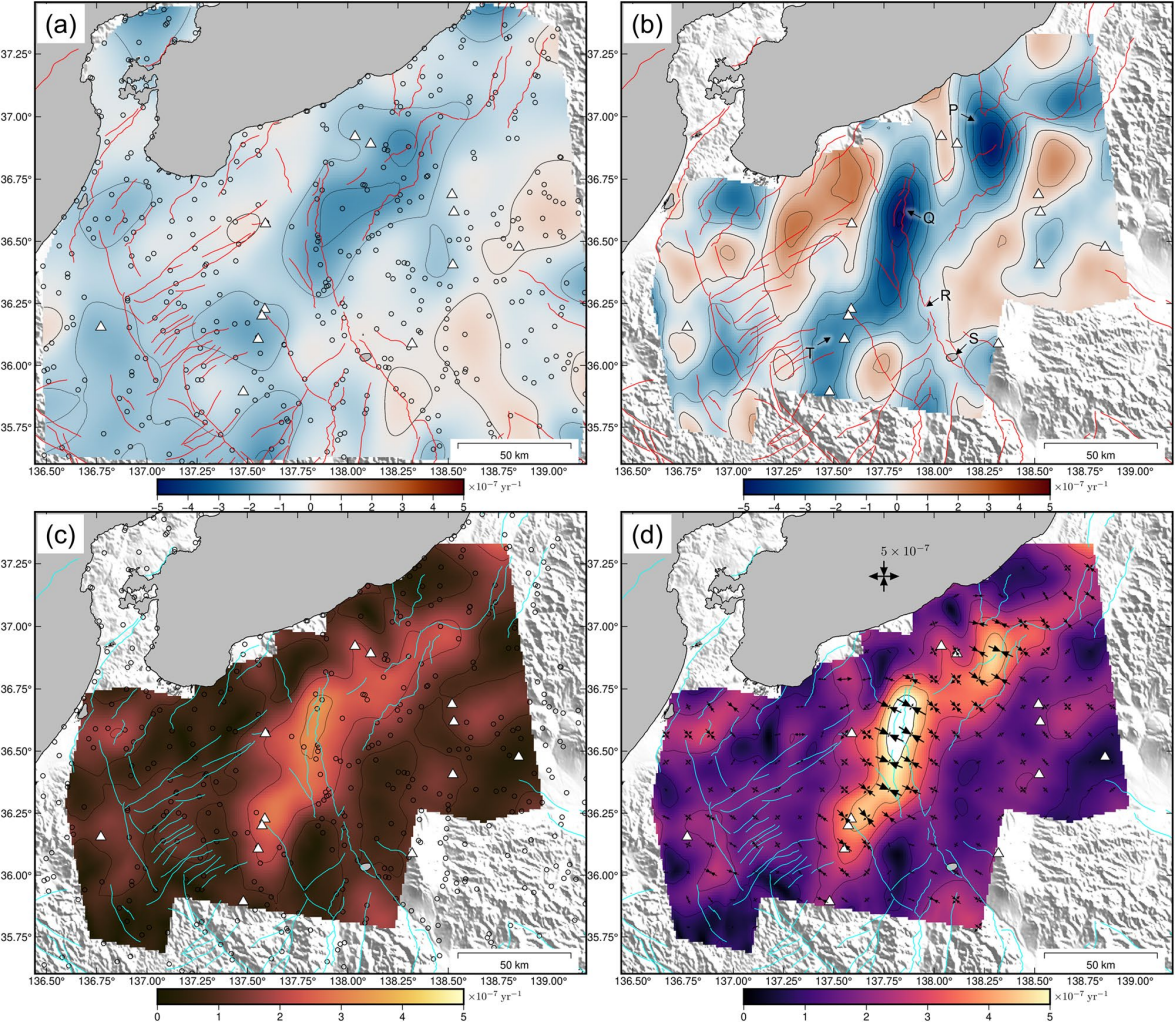
Strain rate invariants. (**a**) Dilatation rate from GNSS only, estimated by a conventional method^47^ with a distance decay constant of $15~\textrm{km}$. (**b**–**d**) Strain rates estimated by InSAR and GNSS. (**b**) Dilatation rate. (**c**) Maximum shear strain rate. (**d**) Second invariant of the strain rate tensor. The radius *R* is set to $15~\textrm{km}$. Open circles mark GNSS stations. Coupled black arrows show magnitudes and orientations of the principal strain rates. White triangles indicate Holocene volcanoes. Red (**a**,**b**) and cyan (**c**,**d**) lines indicate active faults. Contour interval is $1\times 10^{-7}~\mathrm {yr^{-1}}$. Maps in this figure were generated using the Generic Mapping Tools version 6 (https://www.generic-mapping-tools.org/, accessed on 6 April 2026).

Here, *x* and *y* denote the east-west and north-south coordinates, respectively, and *z* denotes eastward or northward velocity components. The coefficients *a* and *b* correspond to the spatial gradients $\frac{\partial z}{\partial x}$ and $\frac{\partial z}{\partial y}$. We then calculated the strain rate tensor and its invariants as shown in Fig. 5 (Supplementary Text S4). The choice of the radius *R* controls the spatial resolution of the derived strain rate field. As shown in Fig. 2a, the average distance between GNSS stations is 8–9 km, and the most values are $<15~\textrm{km}$. Therefore, we adopted $R = 15~\textrm{km}$ to compute the gradient tensor. The individual components of the velocity gradient tensor are shown in Supplementary Fig. S5 (results for other *R* values are given in Supplementary Figs. S6–S8).

## Results

### Interseismic velocity fields

The horizontal velocity field derived from the GNSS observations (Fig. 2b) reveals a deformation pattern characterized by NW–SE convergence. Along the NKTZ (Fig. 1), the velocity vectors exhibit strong spatial variation, implying the presence of a high strain rate. The east–west velocity field derived from InSAR data (Fig. 3a) shows relative eastward motion in the north and westward motion in the south, consistent with the GNSS velocity field. A pronounced velocity change is evident to the north of the NKTZ as well as around the northern part of the ISTL. The latter is highlighted by the kink of the blue contour in Fig. 3a, suggesting concentrated deformation.

The vertical velocity field from GNSS data (Fig. 2c) exhibits broad uplift in the east and subsidence in the west, which reflects postseismic deformation following the 2011 Tohoku-Oki earthquake^44^. In contrast, the vertical velocity field derived from InSAR (Fig. 3b) reveals more localized patterns. In particular, significant uplift is observed in the northern part of the Hida mountain range (A in Fig. 3b, $\sim 6~\mathrm {mm~yr^{-1}}$) and on the eastern side of the northern ISTL (B in Fig. 3b, $\sim 6~\mathrm {mm~yr^{-1}}$). The latter may be associated with the 2014 Northern Nagano earthquake ($M_w$ 6.2); we will further investigate it in the discussion.

In order to validate accuracy of our velocity fields, we also tried a simultaneous inversion method^20,45,46^ which integrates ascending, descending, and GNSS velocities (Supplementary Fig. S9). The resultant 3D velocities are generally consistent with our results, although the small-scale deformations are not observed well, such as the localized uplift in the northern part of the Hida range (A in Fig. 3b), due to the smoothing constraint and sparsity of the triangular mesh.

### Horizontal strain rate maps

In Fig. 5, concentrated strain rates are evident along both the ISTL and the NKTZ (Fig. 1b). The principal strain rate axes (Fig. 5d) indicate NW-SE compression, consistent with the GNSS velocity field (Fig. 2b). Compared to GNSS-only results (Fig. 5a; see also Supplementary Fig. S10), the strain rate fields derived from InSAR and GNSS (Fig. 5b) reveal shorter-wavelength deformation and substantially larger amplitudes, underscoring the higher spatial resolution achieved by incorporating InSAR. Below we describe these features in more detail.

Along the ISTL (Fig. 1b), the strain rate concentration extends north-south direction and correlates with the active faults. This pattern is revealed for the first time through InSAR data. This strain concentration pattern is consistently observed in dilatation ($I_1$, Fig. 5b), maximum shear (Fig. 5c), and the second invariant ($I_2$, Fig. 5d). Particularly high strain rates are observed in the northern ISTL (Q in Fig. 5b), with $|I_1|$, maximum shear, and $I_2$ reaching up to $4\times 10^{-7}~\mathrm {yr^{-1}}$, $3\times 10^{-7}~\mathrm {yr^{-1}}$, and $5\times 10^{-7}~\mathrm {yr^{-1}}$, respectively. In contrast, no significant concentration is detected in the northernmost part of the ISTL. South of Matsumoto (R in Fig. 5b), the strain rates decrease sharply, with maximum shear dropping below $1\times 10^{-7}~\mathrm {yr^{-1}}$ around Lake Suwa (S in Fig. 5b). To the south of latitude 36.25^ο^﻿N (Matsumoto), the strain rate concentration shifts into the southernmost part of Hida mountain range and its southern extension (T in Fig. 5b), where $|I_1|$ and maximum shear reach $2\times 10^{-7}~\mathrm {yr^{-1}}$, and $I_2$ reaches $3\times 10^{-7}~\mathrm {yr^{-1}}$. This localization in the southern extension of Hida mountain range is newly identified in this study and will be discussed in the later section.    

In the northeastern NKTZ (Fig. 1b), strong contraction of up to $4\times 10^{-7}~\mathrm {yr^{-1}}$ is detected east of Mt. Myoko (P in Fig. 5b). This area is dominated by pronounced east-west shortening with relatively low shear strain rates ($<2\times 10^{-7}~\mathrm {yr^{-1}}$), unlike the deformation style of the northern ISTL.

To the east of Hida mountain range, the NKTZ exhibits elevated strain rates. The spatial distribution of $I_1$ shows multiple localized peaks ($1\times 10^{-7}~\text {--}~4\times 10^{-7}~\mathrm {yr^{-1}}$). In contrast, the distribution of maximum shear and $I_2$ are more continuous along the NKTZ, with amplitude of $\sim 3\times 10^{-7}~\mathrm {yr^{-1}}$ and $\sim 4\times 10^{-7}~\mathrm {yr^{-1}}$, respectively (Fig. 5c and d). Since there are no long active faults running through this region, the deformation is likely accommodated by distributed short active faults and folding. Indeed, the mapped active faults (red and cyan lines in Fig. 5) are short, scattered, and show no simple correlation with the magnitude of the strain rates. Closer investigations of active faults^48,49^ further highlight their highly scattered and discontinuous distribution.

On the western side of the Hida mountain range, the NKTZ is characterized by lower strain rates (Fig. 5). Although maximum shear of $<2\times 10^{-7}~\mathrm {yr^{-1}}$ is observed along the Atotsugawa fault (Fig. 1c), the deformation is more diffuse and broadly distributed.

## Discussion

### Relation between the strain rate concentration and geologic structures

**Fig. 6:**
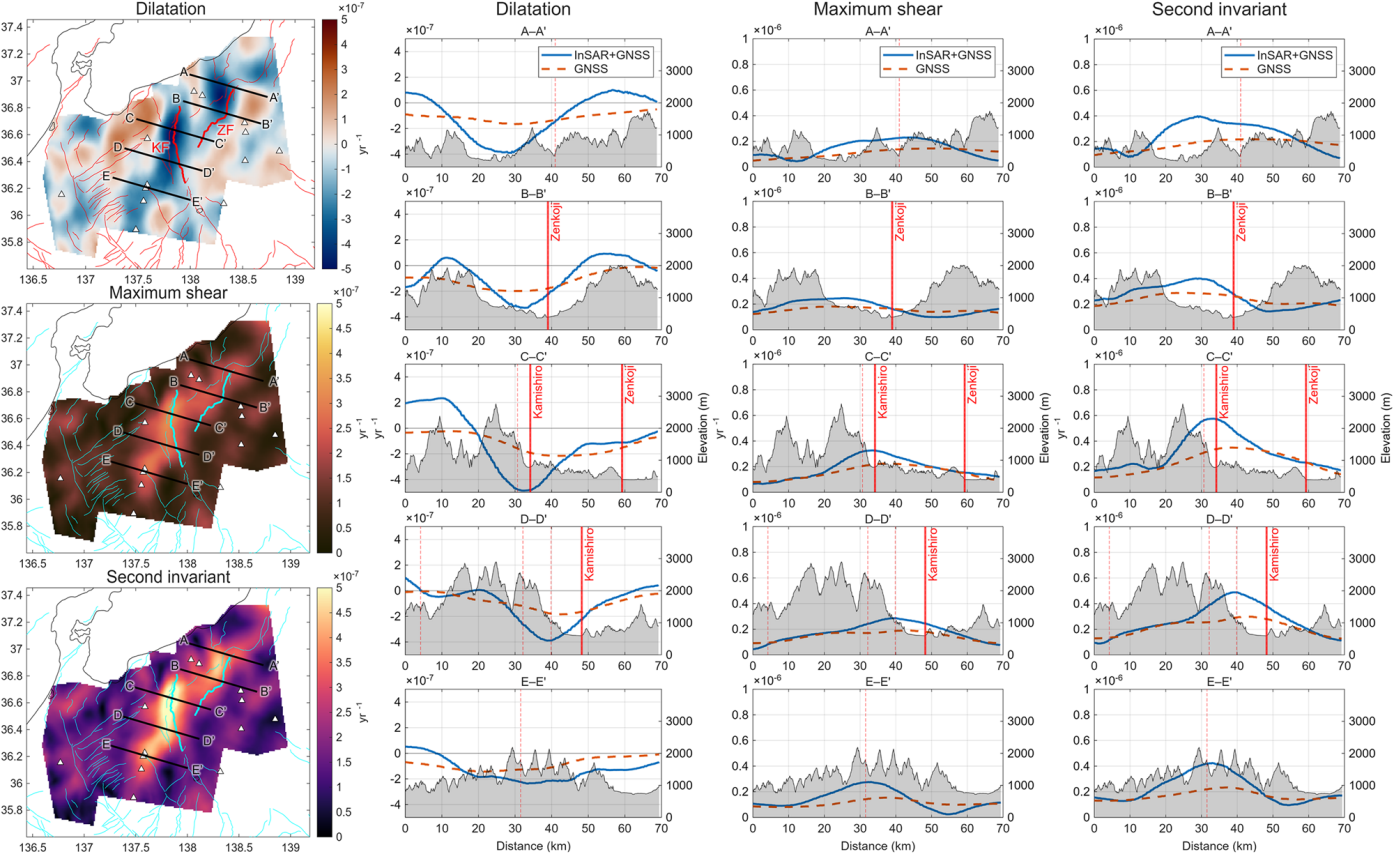
Invariants of the strain rates for representative profiles. The radius *R* is set to 15 km. Left: location of the profiles (A–A’ to E–E’) with the strain rate invariants estimated from InSAR and GNSS (Fig. 5). Red and cyan lines indicate active fault traces, with the Kamishiro fault (KF) and the Zenkoji fault (ZF) highlighted (Fig. 1c). The white triangles denote Holocene volcanoes. Right: strain invariants along the profiles. The blue curve represents strain rate estimated from InSAR and GNSS (Fig. 5b,c,d), and the dotted orange curve indicates strain rate from GNSS alone (Fig. 5a). The vertical red lines denote active faults. The black line with shaded area denotes topographic elevation. Maps in this figure were generated using the MATLAB software R2025b (https://www.mathworks.com/products/matlab.html, accessed on 6 April 2026).

As shown in Fig. 5, the strain rates estimated by InSAR and GNSS are much more localized than those from GNSS alone. Here we compare these strain rates concentrations with known geologic structures (Fig. 1) and discuss possible causes of the strain rate concentration based on representative profiles (Fig. 6).

**Fig. 7:**
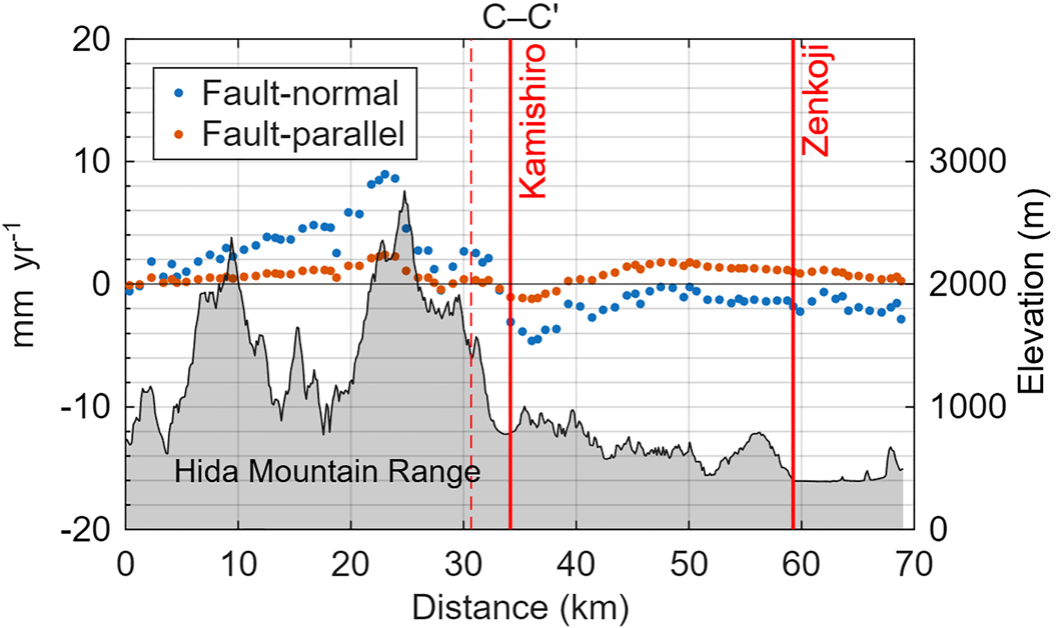
Velocity profile along C–C’ in Fig. 6. Long-wavelength components were removed. Blue and red dots indicate velocity components normal and parallel to the Kamishiro fault, respectively. Red vertical lines indicate active faults. Gray shading shows the topography.

First, we focus on the northern part of the ISTL where two active faults run in parallel: the Kamishiro fault (thick red line in Fig. 6) and a western fault zone consisting of several fault segments^48,49^ (dotted red lines in C–C’ profile of Fig. 6). The strain rates culminate around the traces of these active faults (C–C’ in Fig. 6), indicating that the fault activities reflect to the observed strain rate concentration. The GNSS-only estimates (Fig. 5a) also show elevated strain rates here, consistent with earlier GNSS survey^11^. However, the sharp strain rate localization revealed by InSAR and GNSS (blue lines in Fig. 6) has not been captured by previous GNSS surveys, possibly because the GNSS station coverage is sparse in the mountainous areas (Fig. 1b). Since these two active faults closely locate and run parallel (Fig. 6), it is difficult to resolve the relative contributions solely from the strain rates. Therefore, we check the velocity profile estimated from InSAR and GNSS along C–C’ (Fig. 7). Long-wavelength components were removed to isolate the local deformation. The fault normal component indicates rapid convergence at $\sim 7~\mathrm {mm~yr^{-1}}$ across the Kamishiro fault, which exceeds the estimated long-term slip rates (4.4–$5.4~\mathrm {mm~yr^{-1}}$)^50^. Thus, even considering that the Kamishiro fault is known to be a predominantly east-dipping reverse fault, the slip on the Kamishiro fault at depth alone cannot fully explain the observed velocity pattern. The western fault segments, whose long-term slip rates are poorly constrained, do not associate such rapid horizontal convergence (Fig. 7). The discrepancy between the strain rates and the fault activities may reflect either an underestimated long-term slip rate of the Kamishiro fault or a contribution from unknown geologic structure within the Hida mountain range. As we go south around Matsumoto (D–D’ in Fig. 6), the peak location of the strain rates deviates to the west of the Kamishiro fault, possibly reflecting activities on the western fault segments or unknown blind faults beneath thick sediments^51,52^.

Second, along the southernmost part of the Hida mountain range and its extension (profile E–E’ of Fig. 6), the strain rates are localized in the absence of large active faults but in association with active volcanoes such as Mt. Yake, Mt. Norikura, and Mt. Ontake (Fig. 1c). Considering no large active faults and shallow Curie point depth^53^, the crustal weakening due to high geothermal gradient is a likely cause of this strain rate concentration as previously reported in the northeast Japan^54–57^.

Third, the NKTZ exhibits a broader strain rate peak than the ISTL. In its northeastern part (A–A’ in Fig. 6), many short active faults are distributed across a wide area. Similar distributed deformation has been reported from GNSS surveys in the Niigata plain^17,58^, the northeastern extension of the study area, where intense strain rates were attributed to numerous active faults and folds^59–61^. Along profile B–B’ in Fig. 6, the location of the strain rate peak lies approximately $10~\textrm{km}$ west of the Zenkoji fault, suggesting that deformation in the NKTZ is not accommodated by a single major fault but rather by short, distributed faults and folds that remain insufficiently mapped.

Finally, we discuss the role of active volcanoes, such as Mt. Tateyama, Mt. Myoko, and Mt. Hakusan, which lie along the NKTZ (Fig. 1c). These are active volcanoes but no volcanic-related deformation have been detected by GNSS during the study period^62^. The Atotsugawa fault trace disappears where it intersects Mt. Tateyama and Mt. Hakusan, suggesting that in these volcanic areas, crustal deformation is accommodated by distributed ductile processes rather than brittle faulting under high geothermal conditions^53,63,64^. More broadly, subduction of the oceanic plate influences strain accumulation in central Japan through slab dehydration, volcanic activities, and associated crustal weakening^65^, similar to northeastern Japan^66^. Thus, the causes of strain rate concentration in Japan differ fundamentally from those in non-volcanic regions^18–22^.

### Comparison with seismicity

Numerous earthquakes occurred along the ISTL, the NKTZ, and the Hida mountain range during the study period (Fig. 1c), although most were microearthquakes. The only large event was the 2014 Northern Nagano earthquake, which is discussed in the next section. The dominant focal mechanisms of these earthquakes are strike-slip and reverse faulting, with P-axes oriented in the NW-SE direction^67^. Stress inversion analyses indicate that the maximum compressive stress direction is NW-SE across the study area^67–69^, consistent with the orientations of the estimated principal strain-rate axes (Fig. 5d). The distribution of small earthquakes along the ISTL, the NKTZ, and the Hida mountain range is broadly consistent with that of the high strain-rate zones (Fig. 5). In contrast, seismicity along the southern ISTL, south of Matsumoto and Suwa (R and S in Fig. 5b), is not associated with high strain rates (Fig. 5).

### Effect of postseismic deformation

In the northern part of the ISTL, the 2014 Northern Nagano earthquake ($M_w$ 6.2) occurred on 22 November 2014^39,70^ (black beach ball in Fig. 1c). To exclude the coseismic displacement, we did not use SAR and GNSS data acquired before the earthquake in the hypocentral area (Supplementary Fig. S2). We further examined whether postseismic deformation affected our strain rate estimates. The earthquake ruptured an east-dipping fault plane (black rectangle in Fig. 3b), with a slip of $\sim 1.2$ m in both strike-slip and reverse components reported by previous studies^39^. The velocity field we estimated (Fig. 3b) shows 5–$6~\mathrm {mm~yr^{-1}}$ uplift on the hanging-wall side, which may reflect the postseismic deformation. However, the cumulative LOS displacement time series does not show any transient behavior (Supplementary Fig. S2). Furthermore, GNSS baseline changes across the rupture area indicate that any postseismic transient was short-lived and of small amplitude (Supplementary Fig. S11). We therefore conclude that postseismic deformation has a negligible effect on our strain rate estimates.

### Comparison with the triangulation measurements

Since the early twentieth century, the nation-wide triangulation surveys have been conducted in Japan^71^. Between 1985 and 1989, an additional local trilateration campaign with 8–10 km station spacing revealed significant strain accumulation east of the Hida mountain range during the $\sim 85$ years since 1902^8–10^. In particular, a large compressive strain of $>3\times 10^{-5}$ was detected along the northern ISTL, consistent in both magnitude and orientation with our strain rate field estimates (Q in Fig. 5b). In the northeastern part of the NKTZ, sharp strain localization was documented around the east of Mt. Myoko, extending southward from Naoetsu city (Fig. 1c), in agreement with our results (P in Fig. 5b). No significant deformation was observed in the northern extension of the ISTL, again consistent with our findings.

Overall, the triangulation-derived strain field since 1902 closely matches our strain rate estimates based on InSAR and GNSS, indicating that interseismic crustal deformation has persisted for at least a century. More importantly, the combination of high-precision InSAR and GNSS observations enables us to capture the deformation in mountainous areas that were not covered by the previous triangulation surveys.

### Uncertainties in velocity and strain rate estimation

Firstly, we quantified the GNSS velocity uncertainties, which is listed in Table S3 and Table S4. The velocity uncertainty is mostly less than $0.3~\mathrm {mm~yr^{-1}}$ for the horizontal components and $0.6~\mathrm {mm~yr^{-1}}$ for the vertical component, with modal values of $0.08~\mathrm {mm~yr^{-1}}$ for the horizontal components and $0.26~\mathrm {mm~yr^{-1}}$ for the vertical component. Next, we compared the InSAR and the GNSS velocities to assess uncertainties in our velocity estimates. As shown in Supplementary Figs. (Fig. S3 and S12), the InSAR and GNSS velocities are consistent, with deviations generally below $3~\mathrm {mm~yr^{-1}}$. As for the E-W component, the root-mean-square difference between the InSAR and GNSS velocities is $2.8~\mathrm {mm~yr^{-1}}$. We also checked elevation dependence of the InSAR velocities (Supplementary Fig. S13), and confirmed that topography-related atmospheric phases are well eliminated through our time-series processing.

We further quantified uncertainties in the strain rate field. For the InSAR east-west velocity estimation, phase uncertainties in the interferograms (Supplementary Text S1) were propagated to the velocity estimation step. For the GNSS north-south velocity interpolation, uncertainties at each interpolation grid were evaluated using a variogram model^72^ (Supplementary Text S3). Spatial gradients and their uncertainties were then computed using Equation 2 (Supplementary Text S4). As shown in Supplementary Fig. S14, the resulting uncertainties in the strain rate field are less than $1\times 10^{-7}~\mathrm {yr^{-1}}$, far smaller than the observed peaks of the strain rates (Fig. 5), confirming the precision of our estimates.

We also evaluated the effect of swath gaps in the InSAR velocity fields, which reached up to $\sim 4~\mathrm {mm~yr^{-1}}$ along the edges of the SAR images (Supplementary Fig. S15). To test their impact, we generated an artificial velocity map with a $4~\mathrm {mm~yr^{-1}}$ gap and recomputed strain rates. The resulting artificial strain concentration was at most $2\times 10^{-7}~\mathrm {yr^{-1}}$ (Supplementary Fig. S16). Since the observed strain rate peaks along the Kamishiro fault and east of Mt. Myoko exceed $4\times 10^{-7}~\mathrm {yr^{-1}}$ (P and Q in Fig. 5; A–A’ and C–C’ in Fig. 6), substantially larger than the uncertainties and artificial effect, we interpret these peaks as ongoing crustal deformation, not artifacts.

We further compared strain rate fields with different calculation method: the simultaneous inversion of 3D velocities from ascending and descending LOS and GNSS velocities^20,45,46^ (Supplementary Fig. S9). The results show generally similar pattern to that of our results (Fig. 5). However, the strain rates derived from simultaneous inversion, which requires smoothing constraints, exhibit coarse resolution and smaller peak amplitudes than those obtained with our method. Although the performance of the simultaneous inversion may be improved by optimizing mesh design and smoothing strength, our two-steps approach, in which high-resolution InSAR east–west velocities can be obtained and used directly, is particularly effective to constrain highly concentrated strain rates in central Japan, where east-west velocity gradients are relatively prominent.

## Conclusions

We generated a high-resolution interseismic strain rate map around the inland plate boundary in central Japan, encompassing the geologic boundary along the Itoigawa–Shizuoka Tectonic Line (ISTL), by combining 10 years of L-band InSAR data with a very dense GNSS network, including private-sector stations. Our results show that strain rate concentrations are more sharply localized and reach higher amplitudes than previously estimated from GNSS alone.

Along the northern ISTL, strain rates are strongly localized ($\sim 5\times 10^{-7}~\mathrm {yr^{-1}}$), coinciding with the Kamishiro Fault. Further south, however, the localized deformation deviates from mapped faults and extends into the volcanic zone along the southern extension of the Hida mountain range, where we identified a distinct strain rate peak of $\sim 3\times 10^{-7}~\mathrm {yr^{-1}}$. No major active faults are known in this area, suggesting that the observed deformation may be accommodated by thermally weakened crust associated with high geothermal activity beneath the volcanic zone.

The Niigata–Kobe Tectonic Zone (NKTZ) represents a broader strain rate concentration zone than the ISTL and exhibits more heterogeneous deformation than previously recognized. Multiple localized strain rate peaks are detected east of the Hida mountain range, reaching up to $\sim 4\times 10^{-7}~\mathrm {yr^{-1}}$, while strain rates are lower to the west. Active faults are distributed throughout the region, but their geometry does not correspond to the observed spatial variability in strain rates.

These findings demonstrate that present-day deformation in central Japan does not simply follow the geologic plate boundaries. Strain rate concentrations correspond not only to active faults but also to volcanic chains, a pattern distinct from strain localization in non-volcanic regions. This study is the first to successfully estimate interseismic strain rates using L-band InSAR in the heavily vegetated volcanic arc. Looking ahead, continued L-band missions such as NISAR and ALOS-4 will be indispensable for further improving strain rate accuracy and monitoring deformation in similar tectonic settings.

## Supplementary Information

Supplementary Information.

## Data Availability

ALOS-2/PALSAR-2 Level 1.1 SAR data are shared among the PALSAR Interferometry Consortium to Study our Evolving Land Surface (PIXEL). The data were provided by the Japan Aerospace Exploration Agency (JAXA) under a cooperative research contract with the Earthquake Research Institute, the University of Tokyo. The PALSAR-2 products are owned by JAXA and the Ministry of Economy, Trade and Industry, Japan. GNSS data from the SoftBank observation network are not publicly available because they are provided by SoftBank Corp. and ALES Corp. to the “Consortium to utilize the SoftBank original reference sites for Earth and Space Science” under the associated contract. However, they can be made available from the corresponding author upon reasonable request and with the permission of SoftBank Corp. and ALES Corp. GNSS data from university stations are available from the corresponding author. GNSS data from GSI stations can be downloaded from the corresponding website: (https://terras.gsi.go.jp/). Hypocenter data used in Fig. 1c were obtained from the Japan Meteorological Agency (JMA) unified hypocenter catalog, available at (https://www.data.jma.go.jp/eqev/data/bulletin/index_e.html). The ALOS-2-derived eastward and upward velocity fields presented in Fig. 3, along with their corresponding 1-sigma uncertainty maps are publicly available in Zenodo at DOI: 10.5281/zenodo.19379038 (https://doi.org/10.5281/zenodo.19379038).
