# Supplementary material for: High-resolution strain rate mapping around inland plate boundary within a volcanic arc using L-band InSAR and dense GNSS networks
Source: Sci Rep. 2026 May 7;16:19575. doi: 10.1038/s41598-026-48775-x (PMC13294497; doi:10.1038/s41598-026-48775-x)
Supplement: Supplementary file 1 — Supplementary Information. [file 41598_2026_48775_MOESM1_ESM.pdf]

Supplementary materials for High-resolution strain rate mapping around inland plate boundary within a volcanic arc using L-band InSAR and dense GNSS networks

Shogo Nagaoka*, Youichiro Takada, Takuya Nishimura, Takeshi Sagiya, and Yusaku Ohta
*nagaoka-tsu1999@eis.hokudai.ac.jp

This document consists of four text sections, four supporting tables and sixteen supporting figures:

- Text S1. InSAR time series analysis and uncertainty estimation.
- Text S2. InSAR long-wavelength noise correction.
- Text S3. GNSS data interpolation.
- Text S4. Strain rate estimation.

- Table S1. Details of the SAR data.
- Table S2. Details of the GNSS data processing.
- Table S3. GNSS velocities and 1-sigma uncertainties in m yr^{-1} (GEONET).
- Table S4. GNSS velocities and 1-sigma uncertainties in m yr^{-1} (Universities).

- Figure S1. Time-perpendicular baseline diagram for the InSAR dataset.
- Figure S2. Example of InSAR line-of-sight (LOS) time series.
- Figure S3. InSAR line-of-sight (LOS) velocities after concatenation and GNSS correction.
- Figure S4. Example of GNSS time series.
- Figure S5. Estimated velocity gradients from eastward (InSAR) and northward (GNSS) velocities.
- Figure S6. Strain rates along the profiles ($R = 20$ km).
- Figure S7. Strain rates along the profiles ($R = 10$ km).
- Figure S8. Strain rates along the profiles ($R = 8$ km).
- Figure S9. Three-dimensional velocity and strain rate fields derived from the simultaneous inversion of GNSS and InSAR line-of-sight velocities.
- Figure S10. GNSS strain rates estimated by a conventional method.
- Figure S11. GNSS baseline length change.
- Figure S12. Comparison between InSAR- and GNSS-derived eastward velocities.
- Figure S13. Height-dependence check of the average InSAR velocities.
- Figure S14. Histogram of the estimated standard deviations for the strain rate fields.
- Figure S15. InSAR eastward velocity along the profiles.
- Figure S16. Effect of a swath gap in the strain rate estimates.

S1 InSAR time series analysis and uncertainty estimation

We estimated the InSAR line-of-sight (LOS) velocities and their uncertainties using a weighted least-squares inversion[1,2]. We defined the model vector $\mathbf{m}$ as the cumulative LOS displacement and related it to the data vector $\mathbf{d}$ as follows:

$$\begin{aligned} &\mathbf{d} = \mathbf{G}\mathbf{m}, \\ &G_{i,j} = \begin{cases} -1 & (H_{i,1} = L_j), \\ 1 & (H_{i,2} = L_j), \\ 0 & \text{otherwise}, \end{cases} \end{aligned} \tag{S1}$$

where $\mathbf{d} = \{d_i \mid i = 1, 2, \cdots, N\}$ is the LOS change of the i-th interferogram and $\mathbf{m} = \{m_j \mid j = 1, 2, \cdots, M-1\}$ is the cumulative LOS displacement relative to the first SAR acquisition; N is the number of interferograms and M is the number of SAR acquisitions. $H_{i,1}$ and $H_{i,2}$ denote the primary and secondary acquisition dates of the i-th interferogram, and L_j denotes the end-date of the cumulative displacement m_j, corresponding to the SAR acquisition that terminates the cumulative interval.

We quantified uncertainties of the interferograms using a theoretical standard deviation based on coherence[3,4]:

$$\sigma_\phi = \frac{1}{\sqrt{2N_L}} \frac{\sqrt{1-\gamma^2}}{\gamma}, \tag{S2}$$

where N_L is the effective number of looks (number of independent samples per pixel) and γ is the interferometric coherence. The square of σ_ϕ was assigned to the diagonal elements of the weighting matrix $\mathbf{W}$:

$$[\mathbf{W}]_{i,j} = [\mathrm{Cov}(\mathbf{d})]_{i,j} = \delta_{i,j}\sigma_\phi^2. \tag{S3}$$

The model vector was estimated as follows[5]:

$$\mathbf{m}^{\mathrm{est}} = [\mathbf{G}^{\mathrm{T}}\mathbf{W}\mathbf{G}]^{-1}\mathbf{G}^{\mathrm{T}}\mathbf{W}\mathbf{d}. \tag{S4}$$

The matrix $\mathbf{A}$ was defined as

$$\mathbf{A} = [\mathbf{G}^{\mathrm{T}}\mathbf{W}\mathbf{G}]^{-1}\mathbf{G}^{\mathrm{T}}\mathbf{W}. \tag{S5}$$

Then the covariance matrix of the model vector was obtained as

$$[\mathrm{Cov}(\mathbf{m})] = \mathbf{A}[\mathrm{Cov}(\mathbf{d})]\mathbf{A}^{\mathrm{T}}, \tag{S6}$$

The standard deviations of the cumulative displacements were obtained as the square root of the diagonal elements of $\mathrm{Cov}(\mathbf{m})$. We estimated the LOS velocity by fitting a line to the cumulative displacements (Supplementary Figure S2). We propagated the uncertainties $\mathrm{Cov}(\mathbf{m})$ to the subsequent strain rate calculations (Supplementary Figure S14) in the same way as describe above.

S2 InSAR long-wavelength noise correction

We used GNSS data to correct long-wavelength noise in the InSAR velocity fields[6,7]. First, the 3D GNSS velocities (Figure 2) were projected onto the line-of-sight (LOS) directions of the InSAR measurements. Assuming that the GNSS velocities represent the true ground motion, we treated the difference between the two datasets as the residual noise in the InSAR velocities:

$$z_i^{\mathrm{res}} = z_i^{\mathrm{InSAR}} - z_i^{\mathrm{GNSS}}, \tag{S7}$$

where $z_i^{\mathrm{InSAR}}, z_i^{\mathrm{GNSS}}$ denote the LOS velocities estimated from InSAR and GNSS at the i-th station, respectively, and z_i^{res} is the residual. The residual was modeled as a planar surface:

$$z_i^{\mathrm{res}} = s + px_i + qy_i \tag{S8}$$

where x_i and y_i are the horizontal coordinates of the i-th station. The parameters p, q and s were estimated using a least-squares approach. We then constructed a planar surface that had the same size as the interferogram and subtracted it from all pixels to obtain the corrected InSAR velocities (Supplementary Figure S3).

S3 GNSS data interpolation

To compute spatial gradients from the discrete GNSS velocity observations, we interpolated the GNSS velocities onto a regular grid using the Kriging method[8–10]. We first removed a long-wavelength polynomial surface (drift) from the GNSS north-south velocities (Figure 2b), using a least-squares method. After the trend removal, an experimental variogram $\gamma_E(h)$ was estimated from these velocities:

$$\gamma_E(h) = \frac{1}{2N(h)} \sum_{i=1}^{N(h)} (z_{x_i} - z_{x_i+h})^2, \tag{S9}$$

where z_{x_i} and z_{x_i+h} are the velocities separated by a distance of h, and $N(h)$ is the number of samples in the distance bins. We set the bin interval as 1 km and calculated the average values for every bin. Next, we fit the experimental variogram with a spherical model:

$$\gamma(h) = \begin{cases} b + (c-b)\left(1 - \frac{3}{2}\frac{h}{a} + \frac{1}{2}\frac{h^3}{a^3}\right), & (0 \le h \le a) \\ c, & (h > a) \end{cases} \tag{S10}$$

where a, b, and c are the range, nugget, and sill of the variogram, respectively. We performed the regression and obtained these constants of 8×10^4 m, 1.2×10^{-6} m^2 yr^{-2}, 3.3×10^{-6} m^2 yr^{-2}, respectively.

Using this variogram model, we interpolated the data into the regular grid by the weighted sum of the surrounding velocity data z_k. The weights λ_k, which minimize the expected variance, were determined by solving the following equation:

$$\sum_k \lambda_k C_{j,k} = C_{j,0}, \tag{S11}$$

where $C_{j,k}$, $C_{j,0}$ denote the covariance between the j-th and k-th, and j-th and target point x_0, respectively. The covariance matrix $C_{j,k}$ was constructed from both the variogram model $\gamma(h)$ and the standard deviation σ_k of k-th GNSS velocity[11,12] as follows:

$$C_{j,k} = c - \gamma(h_{j,k}) + \delta_{j,k}\,\sigma_j \sigma_k, \tag{S12}$$

where $h_{j,k}$ is a distance between the j-th and k-th points, and $\delta_{j,k}$ is the Kronecker delta. By solving Equations S11 and S12 for the weights λ_k, we obtained the interpolated velocity z^* and its estimation variance $\mathrm{Var}(z^*)$ at the target point x_0:

$$\begin{aligned} z^* &= \sum_k \lambda_k (z_k - m_0) + m_0, \\ \mathrm{Var}(z^*) &= c - \sum_k \lambda_k C_{k,0}. \end{aligned} \tag{S13}$$

Here, m_0 denotes the average velocity (drift) at x_0, previously estimated as a polynomial surface.

S4 Strain rate estimation

In order to calculate the strain rate tensor in a numerically robust way, we used a multi-point method. First, the eastward and northward velocities u, v within a circular area of radius R were extracted together with their horizontal coordinates x, y. We then fit a planar surface to these data to estimate the velocity gradients (Equation 2) using the weighted least-squares method[5]. In this procedure, the uncertainties of the east-west InSAR velocities (Supplementary Text S1) and the north-south GNSS velocities interpolated by the Kriging method (Supplementary Text S3) were incorporated into the weighting matrix. Then we obtained the velocity gradient tensor (Supplementary Figure S5) and the strain rate tensor:

$$\begin{bmatrix} e_{xx} & e_{xy} \\ e_{xy} & e_{yy} \end{bmatrix} = \begin{bmatrix} \frac{\partial u}{\partial x} & \frac{1}{2}\left(\frac{\partial v}{\partial x} + \frac{\partial u}{\partial y}\right) \\ \frac{1}{2}\left(\frac{\partial v}{\partial x} + \frac{\partial u}{\partial y}\right) & \frac{\partial v}{\partial y} \end{bmatrix}. \tag{S14}$$

Finally, we calculated the strain rate invariants:

$$\begin{aligned} I_1 &= e_{xx} + e_{yy} = e_1 + e_2, \\ I_{max\_shear} &= \sqrt{e_{xy}^2 + \frac{1}{4}(e_{xx} - e_{yy})^2} = \frac{1}{2}(e_1 - e_2), \\ I_2 &= \sqrt{e_{xx}^2 + e_{yy}^2 + 2e_{xy}^2} = \sqrt{e_1^2 + e_2^2}, \end{aligned} \tag{S15}$$

where e_1, e_2 are the maximum and minimum principal strain rates, and I_1, I_{max_shear} and I_2 are dilatation rate, maximum shear strain rate, and second invariant of the strain rate tensor, respectively.

References

1. Berardino, P., Fornaro, G., Lanari, R. & Sansosti, E. A new algorithm for surface deformation monitoring based on small baseline differential SAR interferograms. *IEEE Transactions on Geosci. Remote. Sens.* **40**, 2375–2383, DOI: 10.1109/TGRS.2002.803792 (2002).
2. Schmidt, D. A. & Bürgmann, R. Time-dependent land uplift and subsidence in the Santa Clara valley, California, from a large interferometric synthetic aperture radar data set. *J. Geophys. Res. Solid Earth* **108**, DOI: 10.1029/2002jb002267 (2003).
3. Rodriguez, E. & Martin, J. M. Theory and design of interferometric synthetic aperture radars. In *IEE Proceedings F (Radar and Signal Processing)*, vol. 139, 147–159 (IET, 1992).
4. Hanssen, R. F. *Radar Interferometry: Data Interpretation and Error Analysis*, vol. 2 (Springer Science & Business Media, 2001).
5. Menke, W. *Geophysical Data Analysis: Discrete Inverse Theory* (Academic press, 2018), fourth edn.
6. Fukushima, Y. & Hooper, A. Crustal deformation after 2004 Niigataken-Chuetsu earthquake, central Japan, investigated by persistent scatterer interferometry. *J. Geod. Soc. Jpn.* **57**, 195–214 (2011). In Japanese with English abstract.
7. Takada, Y., Sagiya, T. & Nishimura, T. Interseismic crustal deformation in and around the Atotsugawa fault system, central Japan, detected by InSAR and GNSS. *Earth, Planets Space* **70**, DOI: 10.1186/s40623-018-0801-0 (2018).
8. Chilès, J.-P. & Delfiner, P. *Geostatistics: Modeling Spatial Uncertainty*. Wiley series in probability and mathematical statistics; Applied probability and statistics (Wiley, 1999).
9. Oliver, M. A. & Webster, R. *Basic Steps in Geostatistics: The Variogram and Kriging*. SpringerBriefs in Agriculture (Springer Cham, 2015).
10. Ou, Q. *et al.* Large-scale interseismic strain mapping of the NE Tibetan Plateau from Sentinel-1 interferometry. *J. Geophys. Res. Solid Earth* **127**, DOI: 10.1029/2022JB024176 (2022).
11. Sudhaus, H. & Jónsson, S. Improved source modelling through combined use of InSAR and GPS under consideration of correlated data errors: Application to the June 2000 Kleifarvatn earthquake, Iceland. *Geophys. J. Int.* **176**, 389–404, DOI: 10.1111/j.1365-246X.2008.03989.x (2009).
12. Hamzehpour, N. & Bogaert, P. Improved spatiotemporal monitoring of soil salinity using filtered kriging with measurement errors: An application to the West Urmia Lake, Iran. *Geoderma* **295**, 22–33, DOI: 10.1016/j.geoderma.2017.02.004 (2017).
13. Kobayashi, T., Morishita, Y. & Yarai, H. SAR-revealed slip partitioning on a bending fault plane for the 2014 Northern Nagano earthquake at the northern Itoigawa–Shizuoka tectonic line. *Tectonophysics* **733**, 85–99, DOI: 10.1016/j.tecto.2017.12.001 (2018).
14. Wang, H. & Wright, T. J. Satellite geodetic imaging reveals internal deformation of western Tibet. *Geophys. Res. Lett.* **39**, DOI: 10.1029/2012GL051222 (2012).
15. Wang, H., Wright, T. J., Liu-Zeng, J. & Peng, L. Strain Rate Distribution in South-Central Tibet From Two Decades of InSAR and GPS. *Geophys. Res. Lett.* **46**, 5170–5179, DOI: 10.1029/2019GL081916 (2019).
16. Shen, Z.-K., Jackson, D. D. & Ge, B. X. Crustal deformation across and beyond the Los Angeles basin from geodetic measurements. *JOURNAL OF GEOPHYSICAL RESEARCH* **101**, 957–984 (1996).
17. Sagiya, T., Miyazaki, I. & Tada, T. Continuous GPS array and present-day crustal deformation of Japan. *Pure Appl. Geophys.* **157**, DOI: 10.1007/PL00022507 (2000).

Orbit	Path	Frame	Beam ID (off nadir angle in degree)
Ascending	126	710, 720	RU2_6 (29.1)
Ascending	126	710, 720	RU2_7 (32.4)
Ascending	126	710, 720, 730	RU2_8 (35.4)
Ascending	126	720, 730	RU2_9 (38.2)
Descending	19	2870, 2880	RU2_7 (32.4)
Descending	19	2870, 2880, 2890	RU2_8 (35.4)
Descending	19	2880, 2890	RU2_9 (38.2)
Descending	20	2880, 2890	RU2_7 (32.4)

Table S1. Details of the SAR data.

Name	Period	# of stations	Software	Strategy	Ocean load model	Reference frame
GEONET	2021-01-01<br>2023-12-31	119	GipsyX Ver. 1.4	PPP+AR	FES2014b	ITRF 2014
Universities	2021-01-01<br>2023-12-31	13	GipsyX Ver. 1.4	PPP+AR	FES2014b	ITRF 2014
SB	2021-05-02<br>2023-12-31	315	GipsyX Ver. 2.2	PPP+AR	NAO99b	ITRF 2014

Table S2. Details of the GNSS data processing.

Site ID	Longitude	Latitude	Velocity (m/yr)			Uncertainty (m/yr)		
			East	North	Up	East	North	Up
0044	138.91	36.70	-1.98e-3	3.87e-3	-6.59e-6	7.34e-5	6.42e-5	2.40e-4
0045	138.87	36.26	-9.55e-3	4.54e-3	3.84e-3	6.56e-5	5.81e-5	1.98e-4
0046	137.85	36.50	-1.17e-4	2.15e-3	-2.14e-3	1.03e-4	6.67e-5	2.86e-4
0047	137.94	35.92	-1.20e-2	2.62e-3	-1.74e-4	9.49e-5	7.04e-5	2.85e-4
0051	138.57	37.40	1.74e-2	4.71e-4	1.65e-4	9.73e-5	5.70e-5	1.78e-4
0052	137.49	36.93	1.02e-2	3.06e-5	-1.29e-3	7.06e-5	5.58e-5	1.91e-4
0053	136.89	37.38	1.23e-2	-2.49e-3	-2.10e-3	6.85e-5	6.37e-5	2.05e-4
0054	136.65	36.66	8.97e-3	-1.32e-3	-4.52e-3	5.62e-5	5.41e-5	1.89e-4
0058	137.35	36.14	-6.44e-3	-9.56e-4	-2.07e-3	6.12e-5	6.42e-5	2.20e-4
0059	137.25	35.80	-1.17e-2	6.77e-4	-4.95e-3	6.25e-5	6.48e-5	2.40e-4
0221	138.55	36.51	-6.10e-3	3.75e-3	9.17e-4	7.77e-5	6.88e-5	2.00e-4
0223	139.08	35.99	-1.31e-2	4.68e-3	4.39e-3	6.14e-5	6.07e-5	1.82e-4
0240	138.79	37.31	1.41e-2	2.26e-3	5.84e-3	1.01e-4	7.31e-5	5.68e-4
0241	138.33	37.23	1.37e-2	1.56e-3	1.20e-3	8.63e-5	5.59e-5	1.73e-4
0242	138.93	37.17	1.14e-2	2.57e-3	1.33e-3	9.64e-5	5.99e-5	2.12e-4
0243	138.10	37.16	1.23e-2	6.71e-4	-1.96e-3	8.72e-5	5.96e-5	2.30e-4
0244	138.61	37.08	8.74e-3	1.05e-3	3.55e-4	8.28e-5	5.28e-5	1.84e-4
0245	137.87	37.05	1.13e-2	1.37e-3	-9.62e-4	8.30e-5	5.43e-5	1.96e-4
0246	138.83	36.99	7.50e-3	3.35e-3	5.22e-3	9.43e-5	8.67e-5	2.62e-4
0247	138.20	36.87	7.81e-3	4.31e-3	4.99e-3	1.08e-4	6.55e-5	2.83e-4
0248	137.00	36.74	8.09e-3	-7.16e-5	-2.04e-3	6.60e-5	6.47e-5	1.94e-4
0250	137.44	36.58	4.60e-3	-3.44e-4	-6.22e-5	7.35e-5	7.08e-5	2.62e-4
0251	136.92	36.40	2.95e-3	1.66e-3	-1.62e-3	8.57e-5	8.81e-5	3.21e-4
0253	137.27	37.45	1.19e-2	-8.77e-3	2.60e-2	1.86e-4	1.32e-4	5.31e-4
0256	136.63	36.17	2.17e-3	-5.14e-4	2.79e-3	7.96e-5	8.92e-5	3.62e-4
0258	136.50	35.98	-2.44e-3	7.00e-4	-3.47e-3	6.76e-5	7.08e-5	2.23e-4
0263	138.32	35.86	-1.38e-2	3.80e-3	1.85e-3	9.06e-5	7.25e-5	2.15e-4
0264	138.70	35.75	-1.26e-2	-3.07e-3	-1.36e-3	7.34e-5	1.64e-4	2.30e-4
0265	138.44	36.80	2.54e-3	2.92e-3	1.02e-3	7.16e-5	5.97e-5	2.60e-4

Table S3 continued from previous page

0266	137.87	36.71	6.41e-3	1.07e-3	-1.35e-3	1.50e-4	6.93e-5	3.54e-4
0267	138.25	36.67	-5.26e-4	3.95e-3	2.14e-4	7.05e-5	5.47e-5	1.85e-4
0268	138.32	36.39	-5.93e-3	4.36e-3	-6.38e-4	8.99e-5	6.57e-5	2.00e-4
0269	138.64	36.35	-8.75e-3	5.36e-3	8.66e-4	7.14e-5	6.46e-5	1.81e-4
0270	137.90	36.32	-4.94e-3	3.66e-3	-9.32e-4	8.86e-5	6.44e-5	2.70e-4
0271	138.22	36.21	-8.82e-3	4.63e-3	3.07e-4	9.30e-5	6.80e-5	2.77e-4
0272	138.46	36.13	-1.03e-2	4.06e-3	-3.97e-3	9.12e-5	8.58e-5	2.73e-4
0273	137.98	36.12	-1.02e-2	2.87e-3	-2.42e-4	7.25e-5	6.62e-5	2.30e-4
0274	137.70	35.78	-1.32e-2	3.76e-3	-1.94e-3	7.28e-5	6.96e-5	2.91e-4
0279	137.15	36.34	3.02e-4	-1.51e-5	5.93e-3	7.46e-5	7.60e-5	3.68e-4
0280	136.95	36.03	-6.35e-3	7.23e-4	-3.37e-3	6.38e-5	6.26e-5	2.50e-4
0281	137.53	35.97	-9.75e-3	1.41e-3	-2.90e-3	6.81e-5	6.70e-5	2.04e-4
0282	136.86	35.87	-7.38e-3	1.19e-3	-4.28e-3	7.09e-5	5.96e-5	1.91e-4
0283	136.98	35.76	-1.03e-2	1.60e-3	-3.28e-3	5.88e-5	5.92e-5	1.97e-4
0284	137.42	35.66	-1.29e-2	3.15e-3	-6.18e-4	5.73e-5	5.97e-5	1.83e-4
0285	136.49	35.64	-7.10e-3	-5.43e-4	-5.13e-3	9.68e-5	1.12e-4	2.76e-4
0286	136.61	35.63	-8.23e-3	4.44e-4	-2.73e-3	6.76e-5	6.44e-5	2.16e-4
0287	137.17	35.61	-1.27e-2	2.79e-3	-5.46e-4	5.55e-5	6.03e-5	2.04e-4
0567	138.52	37.35	1.60e-2	1.05e-3	1.70e-3	9.46e-5	5.94e-5	1.98e-4
0568	139.02	37.32	1.50e-2	1.56e-3	5.81e-4	1.06e-4	6.04e-5	2.19e-4
0569	138.24	37.06	1.04e-2	9.14e-4	2.98e-3	9.58e-5	6.81e-5	2.75e-4
0570	137.89	36.95	1.04e-2	1.51e-3	2.28e-3	9.08e-5	6.67e-5	2.54e-4
0572	137.37	36.74	7.89e-3	3.29e-4	-2.08e-3	6.71e-5	5.43e-5	1.91e-4
0573	137.03	36.65	7.05e-3	1.09e-3	-1.95e-3	6.30e-5	6.46e-5	2.03e-4
0574	137.14	37.31	1.10e-2	-4.92e-3	3.91e-3	6.80e-5	5.88e-5	2.57e-4
0575	136.72	37.16	1.14e-2	-2.06e-3	-2.69e-3	6.07e-5	5.03e-5	1.81e-4
0576	137.00	37.12	1.10e-2	-1.45e-3	-2.56e-3	6.26e-5	5.71e-5	1.84e-4
0577	136.76	36.82	9.44e-3	-7.21e-4	-3.27e-3	6.13e-5	5.01e-5	1.70e-4
0578	136.61	36.37	4.57e-3	-3.73e-4	-3.33e-3	6.13e-5	5.93e-5	2.14e-4
0591	138.59	36.62	-3.78e-3	3.09e-3	6.95e-3	1.17e-4	7.51e-5	3.18e-4
0592	139.07	36.23	-1.07e-2	5.57e-3	8.25e-4	6.97e-5	6.56e-5	3.13e-4
0593	138.91	36.14	-1.08e-2	2.90e-3	1.04e-2	6.42e-5	6.80e-5	3.06e-4
0606	138.69	35.65	-1.64e-2	4.01e-3	2.25e-3	6.23e-5	5.96e-5	2.08e-4
0608	137.98	36.24	-8.04e-3	4.18e-3	-1.14e-3	6.93e-5	6.43e-5	2.27e-4
0609	137.87	36.23	-3.13e-3	-5.98e-4	-5.71e-3	1.42e-4	1.48e-4	2.81e-4
0610	138.50	36.28	-8.29e-3	7.34e-3	-4.40e-3	6.51e-5	8.43e-5	1.97e-4
0611	137.68	36.08	-1.05e-2	2.49e-3	-1.11e-3	1.05e-4	8.22e-5	3.00e-4
0612	138.21	36.03	-1.15e-2	4.47e-3	-5.14e-4	6.37e-5	6.57e-5	2.11e-4
0613	138.58	35.97	-1.31e-2	4.78e-3	2.14e-3	9.02e-5	8.33e-5	2.49e-4
0614	137.60	35.88	-1.03e-2	1.19e-3	1.41e-3	9.46e-5	8.12e-5	2.70e-4
0615	137.98	35.73	-1.29e-2	2.05e-3	-1.48e-3	9.47e-5	8.78e-5	2.74e-4
0617	136.90	36.26	-3.52e-4	1.48e-3	-1.24e-3	8.61e-5	9.46e-5	2.94e-4
0618	137.36	36.29	-3.03e-3	-9.95e-4	-1.83e-3	6.22e-5	7.41e-5	1.83e-4
0619	137.20	35.91	-1.02e-2	3.99e-4	8.80e-4	6.17e-5	6.06e-5	2.37e-4
0752	139.06	36.54	-1.89e-3	6.32e-4	5.36e-4	7.34e-5	8.70e-5	1.74e-4
0754	139.18	35.88	-1.35e-2	4.92e-3	3.19e-3	6.86e-5	6.89e-5	2.60e-4
0802	138.94	36.78	1.26e-3	4.07e-3	3.02e-3	1.01e-4	8.35e-5	2.65e-4
0809	138.46	35.67	-1.17e-2	6.99e-3	3.10e-4	9.69e-5	7.57e-5	2.66e-4
0952	139.06	36.84	3.38e-3	3.62e-3	3.03e-3	1.07e-4	7.76e-5	2.87e-4
0954	138.85	36.59	-3.53e-3	4.22e-3	3.17e-3	6.75e-5	5.69e-5	1.79e-4
0955	139.02	36.40	-7.48e-3	6.43e-3	-3.51e-3	1.01e-4	6.61e-5	2.26e-4
0957	138.70	36.20	-8.43e-3	5.25e-3	3.69e-3	7.38e-5	6.49e-5	2.70e-4
0958	138.93	35.93	-1.28e-2	4.27e-3	4.57e-3	1.10e-4	7.81e-5	3.25e-4
0962	138.63	37.21	1.36e-2	1.51e-3	1.18e-3	1.02e-4	7.64e-5	3.12e-4

Table S3 continued from previous page

0964	138.46	37.11	1.16e-2	1.92e-3	3.40e-3	8.95e-5	6.33e-5	2.34e-4
0966	137.03	36.92	1.06e-2	-5.86e-4	-3.11e-3	6.86e-5	5.78e-5	2.43e-4
0967	137.55	36.86	9.86e-3	-2.38e-4	-1.27e-3	7.48e-5	5.52e-5	2.32e-4
0968	137.15	36.76	8.52e-3	5.44e-4	-1.84e-3	6.68e-5	5.20e-5	2.05e-4
0969	136.81	36.59	6.41e-3	-1.02e-3	-5.54e-3	6.71e-5	7.16e-5	2.50e-4
0970	137.24	36.47	2.56e-3	7.16e-4	2.88e-5	9.96e-5	9.48e-5	3.77e-4
0971	137.03	37.43	9.90e-3	-5.28e-3	-1.16e-3	7.34e-5	7.96e-5	2.54e-4
0972	136.91	37.23	1.16e-2	-2.48e-3	-4.96e-4	7.63e-5	5.42e-5	2.53e-4
0973	136.54	36.54	6.95e-3	-1.09e-3	-5.75e-3	5.63e-5	4.99e-5	1.98e-4
0976	136.66	35.90	-7.29e-3	9.09e-4	-7.06e-3	9.11e-5	8.52e-5	3.00e-4
0979	138.44	35.90	-1.35e-2	4.68e-3	2.32e-3	8.48e-5	7.17e-5	2.94e-4
0982	138.62	36.86	3.38e-3	5.15e-3	4.33e-3	1.05e-4	8.05e-5	3.12e-4
0983	138.10	36.71	3.11e-3	3.03e-3	8.23e-4	7.89e-5	6.27e-5	2.05e-4
0984	138.12	36.52	-4.15e-3	4.84e-3	-6.96e-4	6.81e-5	6.20e-5	2.03e-4
0985	138.37	36.26	-8.45e-3	5.64e-3	1.39e-5	6.64e-5	6.88e-5	1.96e-4
0986	137.79	35.94	-1.08e-2	2.83e-3	-1.14e-3	7.58e-5	6.31e-5	2.67e-4
0987	138.10	35.88	-1.33e-2	3.32e-3	6.14e-4	7.27e-5	6.31e-5	2.59e-4
0988	137.45	35.82	-1.17e-2	2.32e-3	-2.44e-3	6.95e-5	7.37e-5	2.57e-4
0989	137.62	35.66	-1.61e-2	5.92e-3	-2.05e-3	1.02e-4	6.87e-5	2.32e-4
0990	137.08	36.14	-4.01e-3	7.92e-4	-4.45e-3	7.07e-5	7.12e-5	2.60e-4
0991	136.78	35.72	-8.55e-3	1.48e-3	-8.87e-3	6.49e-5	6.65e-5	2.84e-4
1099	137.55	36.11	-8.17e-3	-2.58e-3	-2.16e-3	1.47e-4	1.62e-4	3.16e-4
1127	137.81	35.78	-1.36e-2	3.91e-3	-1.32e-3	8.79e-5	6.86e-5	2.09e-4
1138	137.60	36.58	3.50e-3	-3.48e-4	-1.16e-4	1.15e-4	7.32e-5	2.27e-4
1158	136.77	37.00	1.05e-2	-1.77e-3	-2.34e-3	5.87e-5	5.46e-5	1.65e-4
1206	138.95	35.75	-1.21e-2	5.24e-3	1.14e-3	9.20e-5	6.80e-5	2.38e-4
1238	137.21	36.59	5.45e-3	6.83e-4	-1.19e-3	7.36e-5	6.32e-5	1.97e-4
3064	138.98	35.62	-1.70e-2	2.83e-3	3.80e-2	8.13e-5	9.82e-5	6.33e-4
3065	139.16	35.61	-1.89e-2	5.06e-3	3.52e-2	6.91e-5	6.98e-5	5.98e-4
R001	137.78	36.52	3.51e-3	2.95e-3	-1.07e-2	2.99e-4	2.46e-4	1.04e-3
R003	137.94	36.47	-3.30e-3	7.62e-3	-9.49e-3	1.80e-4	2.31e-4	9.27e-4
R004	137.96	36.46	-4.85e-3	5.38e-3	1.90e-3	8.77e-5	6.77e-5	3.12e-4
R005	138.01	36.44	-3.29e-3	7.56e-3	-8.59e-4	2.89e-4	2.57e-4	9.84e-4
R006	138.05	36.46	-5.57e-3	5.15e-3	-7.35e-4	8.52e-5	7.26e-5	2.50e-4
R015	137.72	36.56	2.66e-3	2.11e-3	5.90e-3	1.34e-4	9.69e-5	4.22e-4
R019	138.06	36.20	-8.69e-3	4.22e-3	-1.50e-3	7.80e-5	7.20e-5	2.74e-4

Table S3. GNSS velocities and 1-sigma uncertainties in m yr^{-1} (GEONET).

Site ID	Longitude	Latitude	Velocity (m/yr)			Uncertainty (m/yr)		
			East	North	Up	East	North	Up
HGSD	137.44	36.42	-1.65e-3	-9.20e-4	-3.69e-3	2.20e-4	1.82e-4	7.32e-4
HIDA	137.31	36.25	-3.53e-3	-3.26e-4	-2.51e-3	1.73e-4	7.87e-5	2.88e-4
HURU	137.19	36.24	-3.25e-3	3.14e-4	-3.88e-3	8.39e-5	7.41e-5	2.46e-4
INAK	137.10	36.26	-1.99e-3	5.54e-4	-2.58e-3	8.38e-5	1.04e-4	3.51e-4
MAKI	137.29	36.40	0.00e+0	0.00e+0	0.00e+0	9.01e-5	8.37e-5	3.79e-4
MNNM	137.12	36.38	1.02e-3	2.86e-4	-2.82e-3	1.18e-4	1.51e-4	4.72e-4
MSSA	138.35	36.14	-1.16e-2	4.11e-3	1.80e-3	1.29e-4	1.34e-4	3.26e-4
NIRE	137.23	36.53	5.84e-3	5.42e-4	2.17e-4	2.07e-4	1.10e-4	5.34e-4
NTYD	137.09	37.37	1.02e-2	-4.71e-3	-6.66e-4	2.16e-4	1.10e-4	3.24e-4
OMKR	137.12	36.30	-1.80e-3	1.96e-4	-2.38e-3	8.03e-5	7.10e-5	2.60e-4
SAKA	137.01	36.43	3.04e-3	1.08e-3	-3.88e-4	1.38e-4	8.67e-5	3.41e-4
USUD	138.36	36.13	-1.07e-2	4.63e-3	2.85e-3	1.04e-4	1.49e-4	4.22e-4
WARI	137.28	36.37	-1.10e-3	-2.41e-4	-3.13e-3	1.97e-4	2.70e-4	5.35e-4

Table S4. GNSS velocities and 1-sigma uncertainties in m yr^{-1} (Universities).

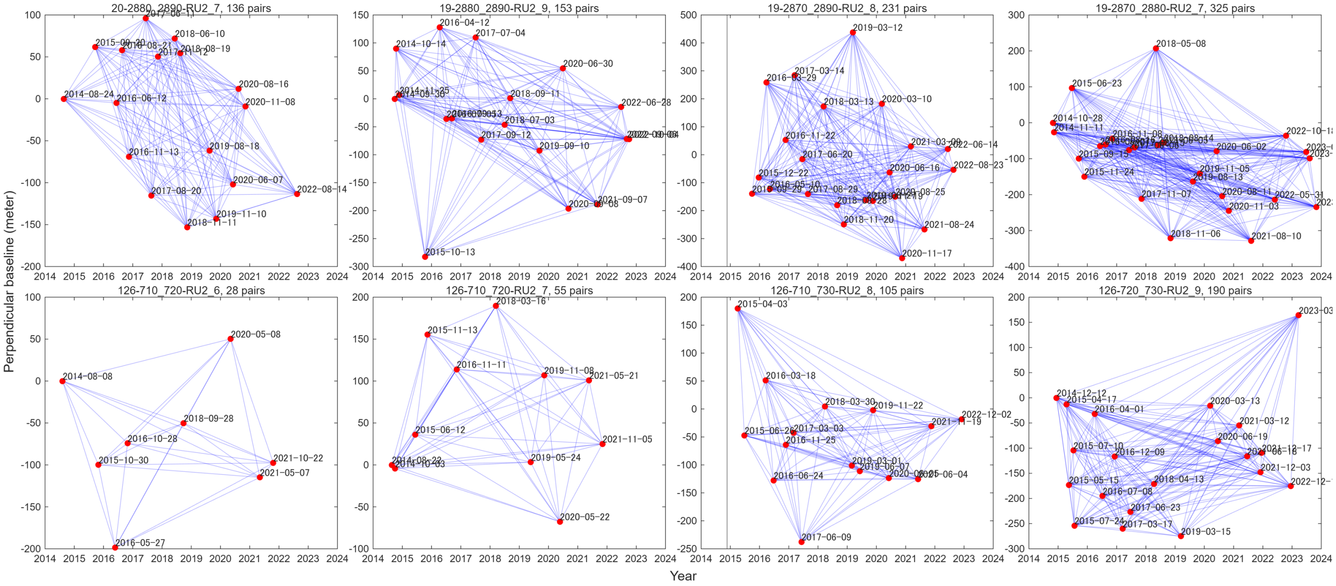

Figure S1. Time-perpendicular baseline diagram for the InSAR dataset. The red dots indicate data acquisition dates. The blue lines denote the interferograms used in this study. The vertical lines indicate the date of the 2014 Northern Nagano earthquake (Figure 1c).

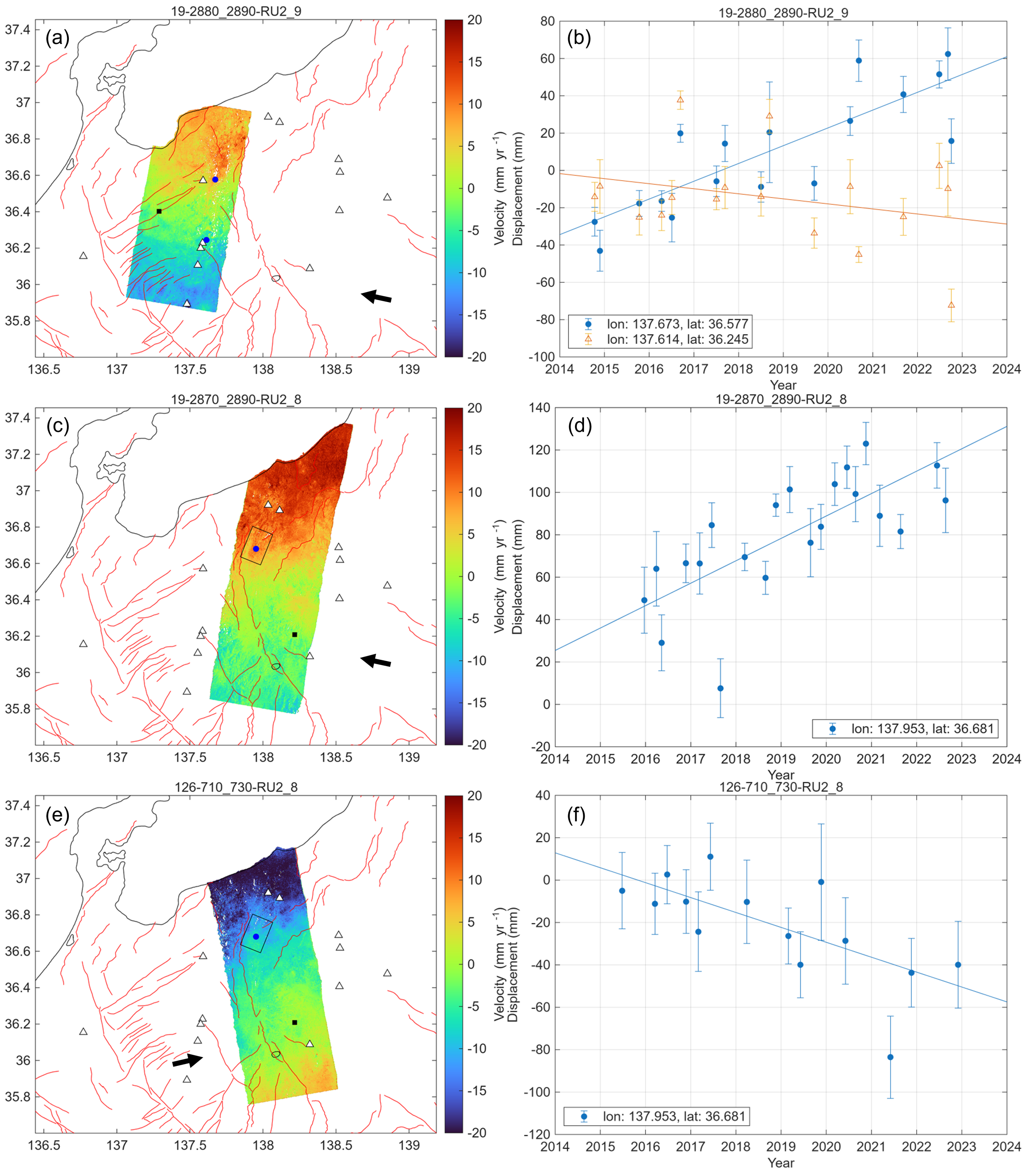

Figure S2. Example of InSAR line-of-sight (LOS) time series. (**a**,**c**,**e**) Average velocities toward the satellite. The black dot indicates the reference point and the black arrow shows the satellite LOS direction. The white triangles indicate Holocene volcanoes and the red lines represent active fault traces. The black rectangle in (**c**,**e**) shows the location of the fault model of the 2014 Northern Nagano earthquake[13]. (**b**,**d**,**f**) LOS time series at the blue dots shown in (**a**,**c**,**e**). The error bars represent standard deviations (Supplementary Text S1). The slope of the fitted line corresponds to the average velocities plotted in (**a**,**c**,**e**). Details of the data are provided in Supplementary Table S1. Maps in this figure were generated using the MATLAB software R2025b (https://www.mathworks.com/products/matlab.html, accessed on 6 April 2026).

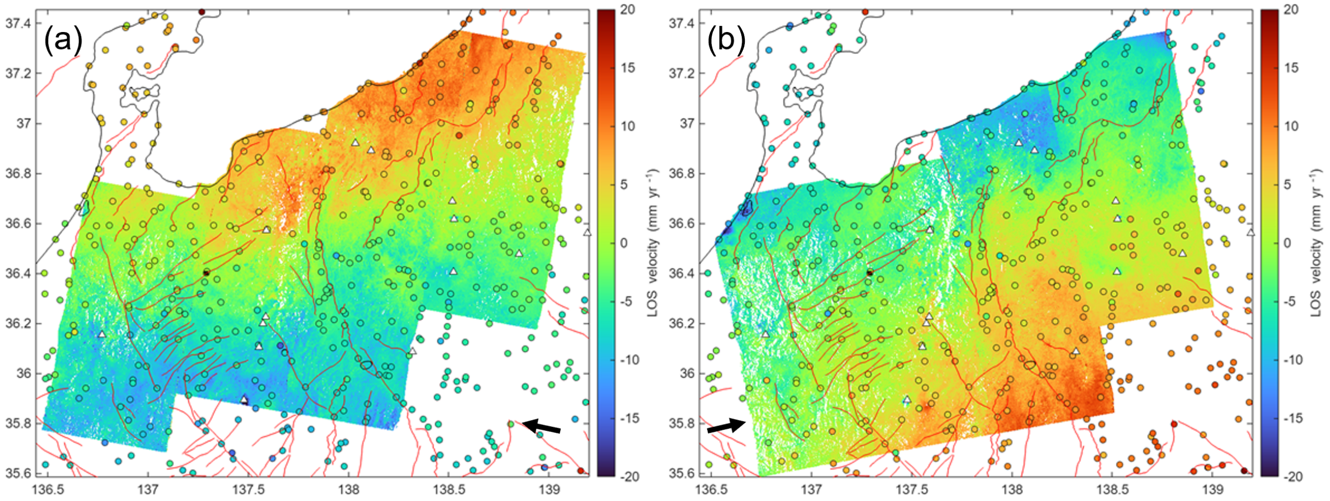

Figure S3. InSAR line-of-sight (LOS) velocities after concatenation and GNSS correction (Supplementary Text S2). (**a**) Descending tracks. (**b**) Ascending tracks. Positive values indicate velocity toward satellite. The colored circles indicate GNSS velocities projected onto the same LOS direction. The black dot indicates the reference point. The black arrow shows the satellite LOS direction. The white triangles indicate Holocene volcanoes and the red lines represent active fault traces. Maps in this figure were generated using the MATLAB software R2025b (https://www.mathworks.com/products/matlab.html, accessed on 6 April 2026).

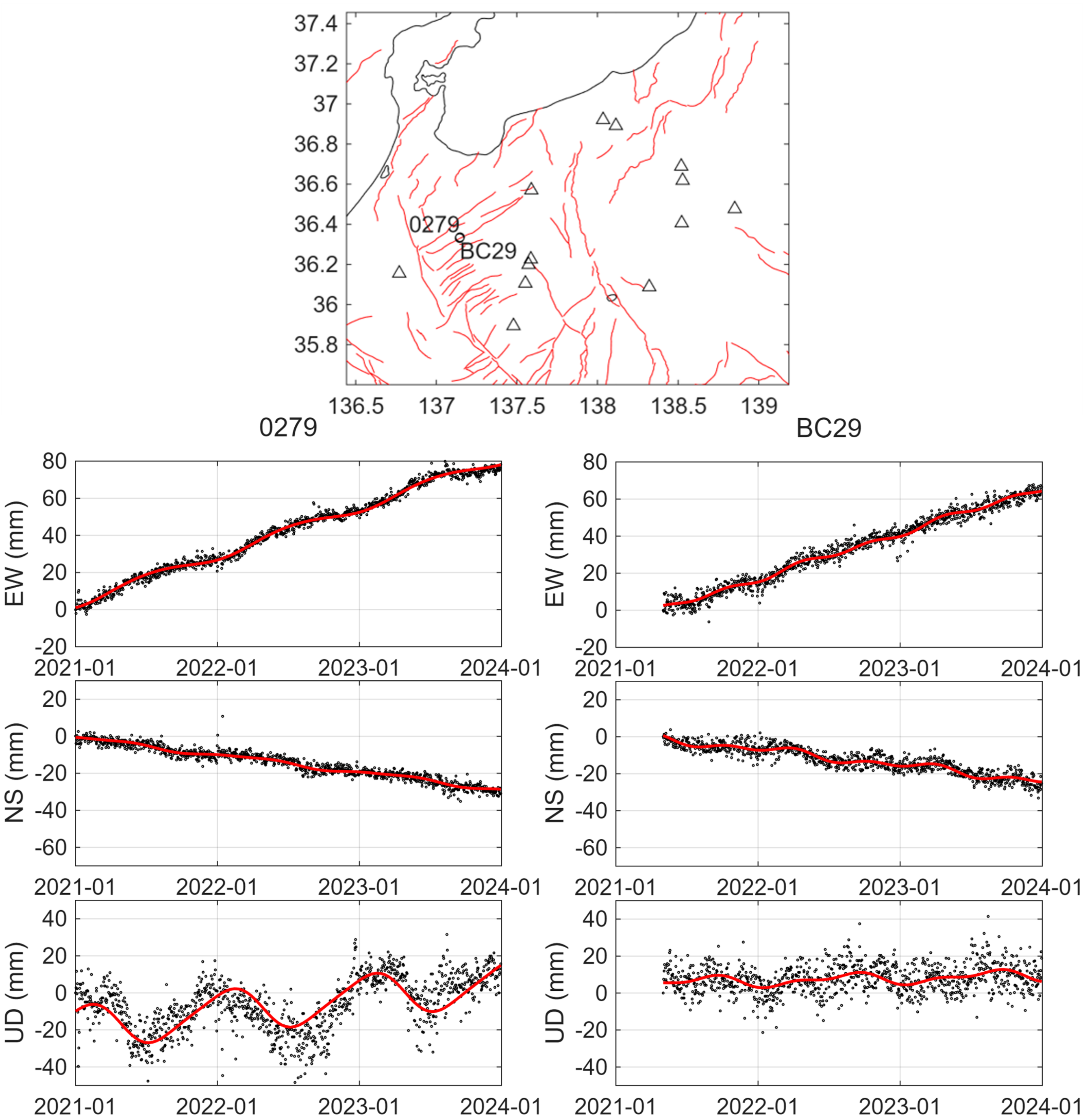

Figure S4. Example of GNSS time series. Top panel shows the location of the stations: 0279 (GEONET) and BC29 (SB). Distance between the stations is < 600 m. Bottom panels show the time series of 0279 and BC29. The red lines indicate the regression results (Equation 1). The map was generated using the MATLAB software R2025b (https://www.mathworks.com/products/matlab.html, accessed on 6 April 2026).

Radius: 15 km

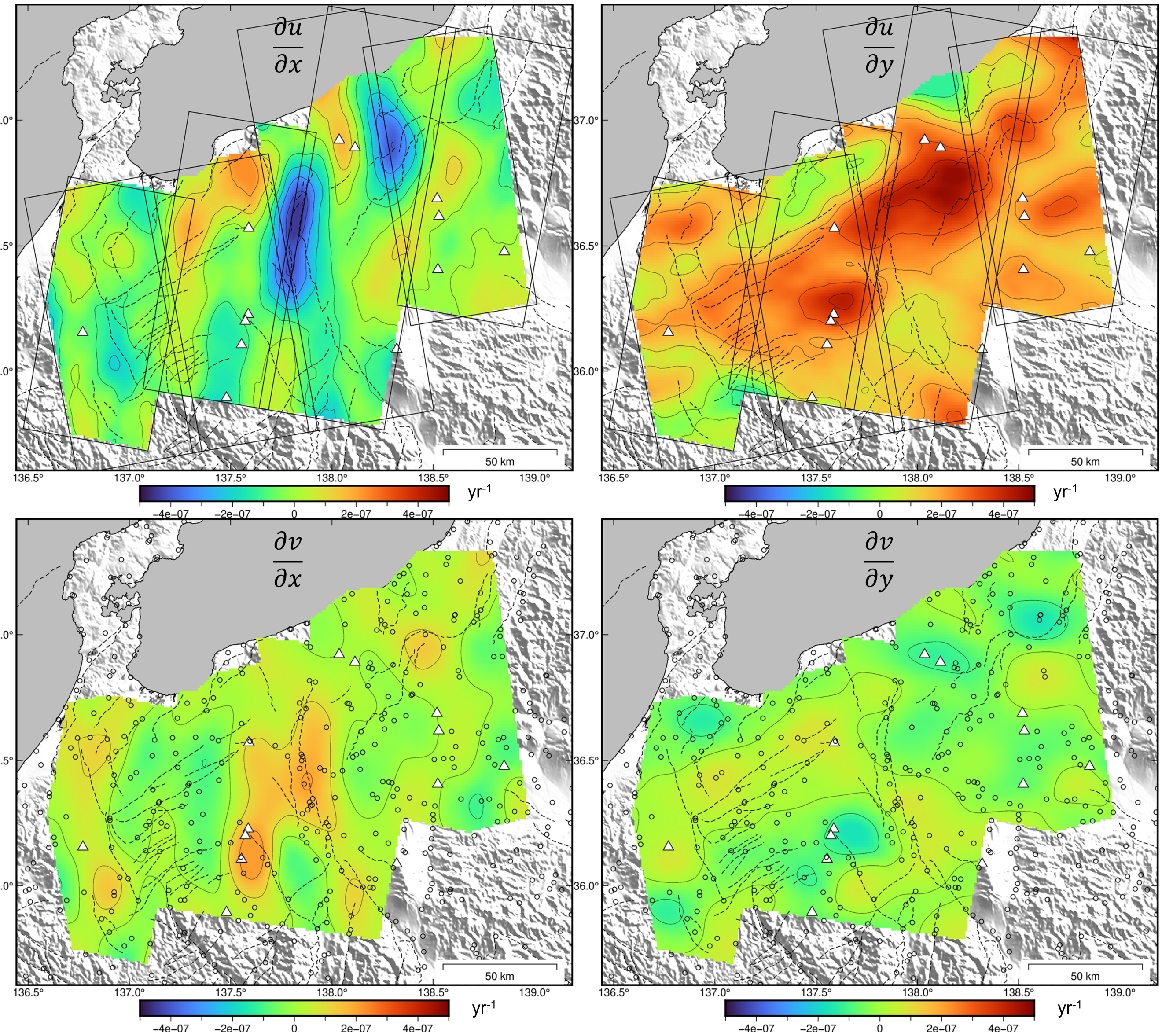

Figure S5. Estimated velocity gradients from eastward (InSAR) and northward (GNSS) velocities (u, v). The east-west and north-south coordinates are indicated as x, y, respectively. The radius R is set to 15 km. Maps in this figure were generated using the Generic Mapping Tools version 6 (https://www.generic-mapping-tools.org/, accessed on 6 April 2026).

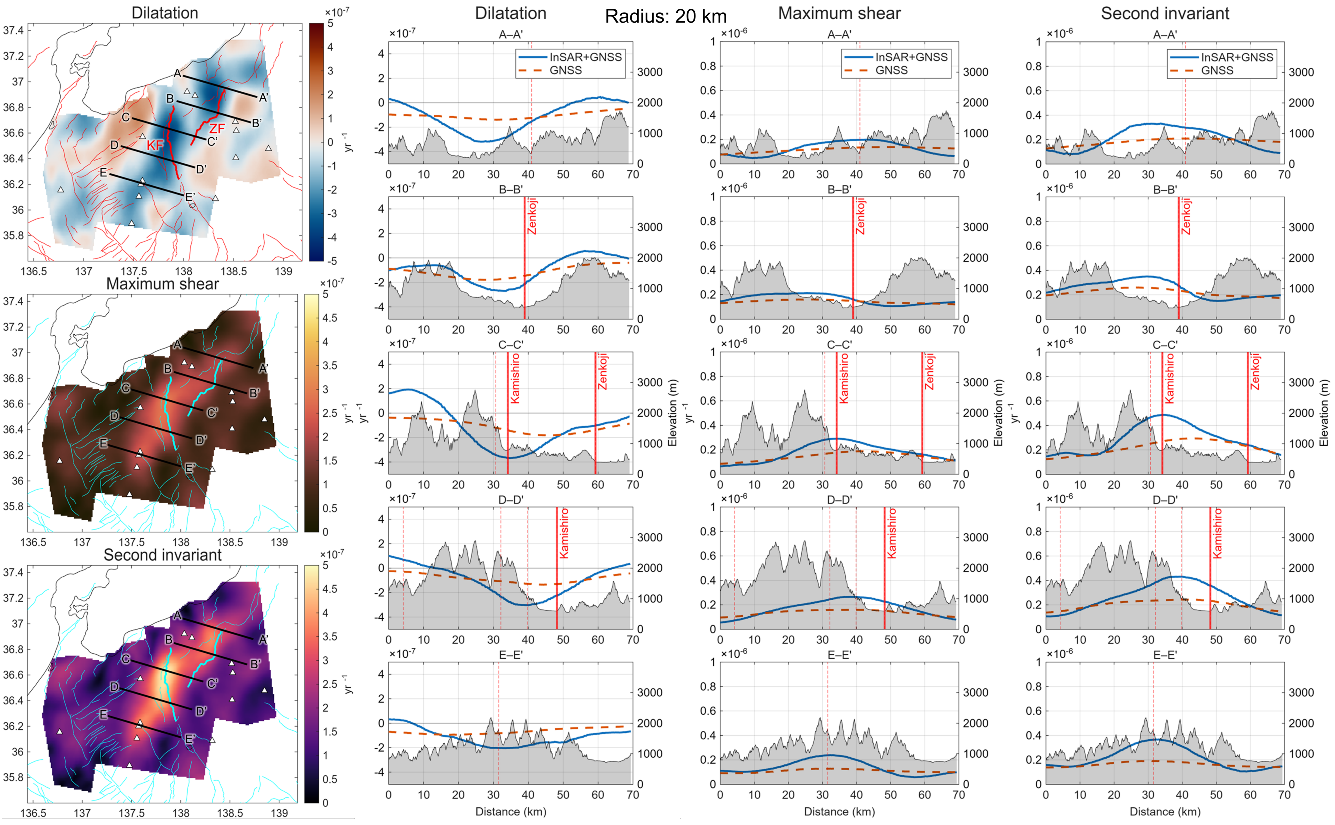

Figure S6. Strain rates along the profiles ($R = 20$ km). Descriptions are the same as Figure 6.

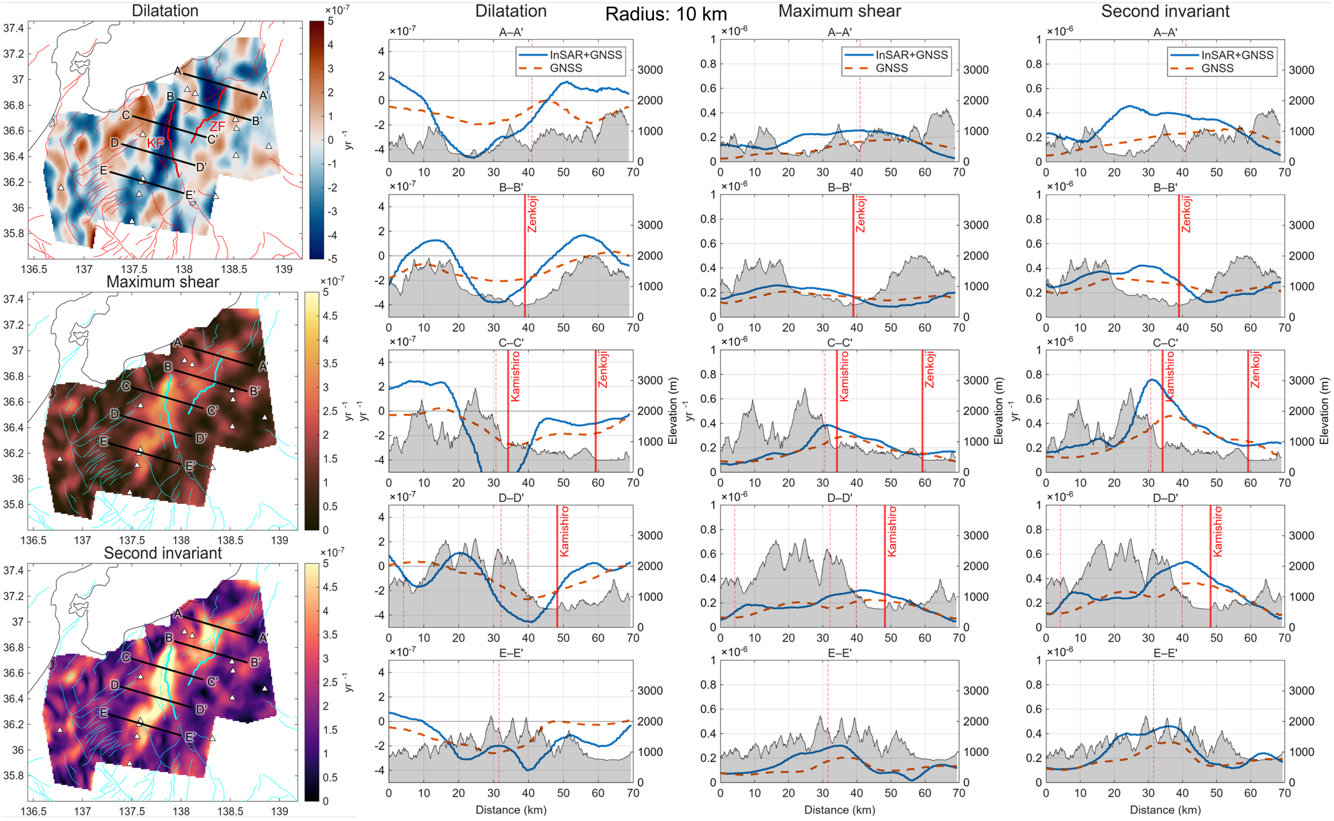

Figure S7. Strain rates along the profiles ($R = 10$ km). Descriptions are the same as Figure 6.

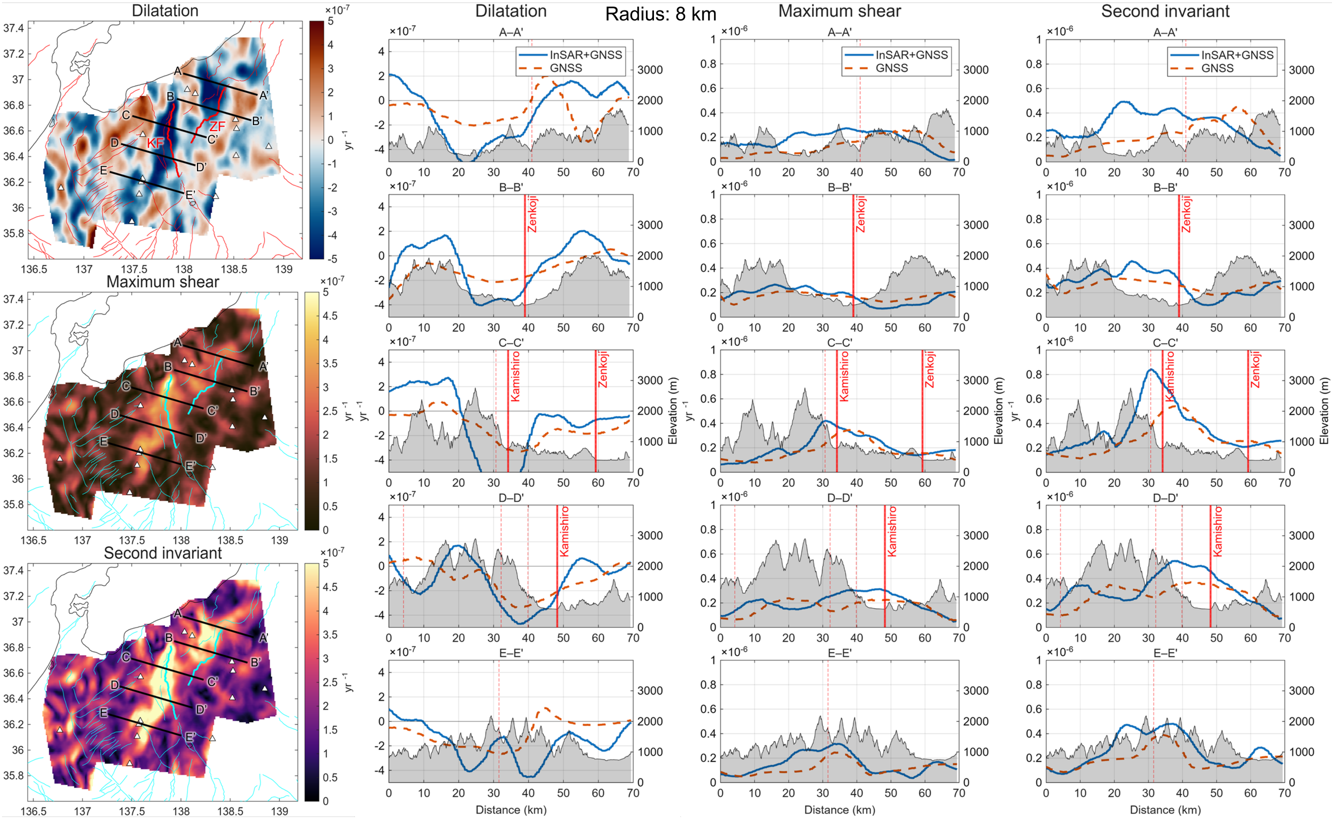

Figure S8. Strain rates along the profiles ($R = 8$ km). Descriptions are the same as Figure 6.

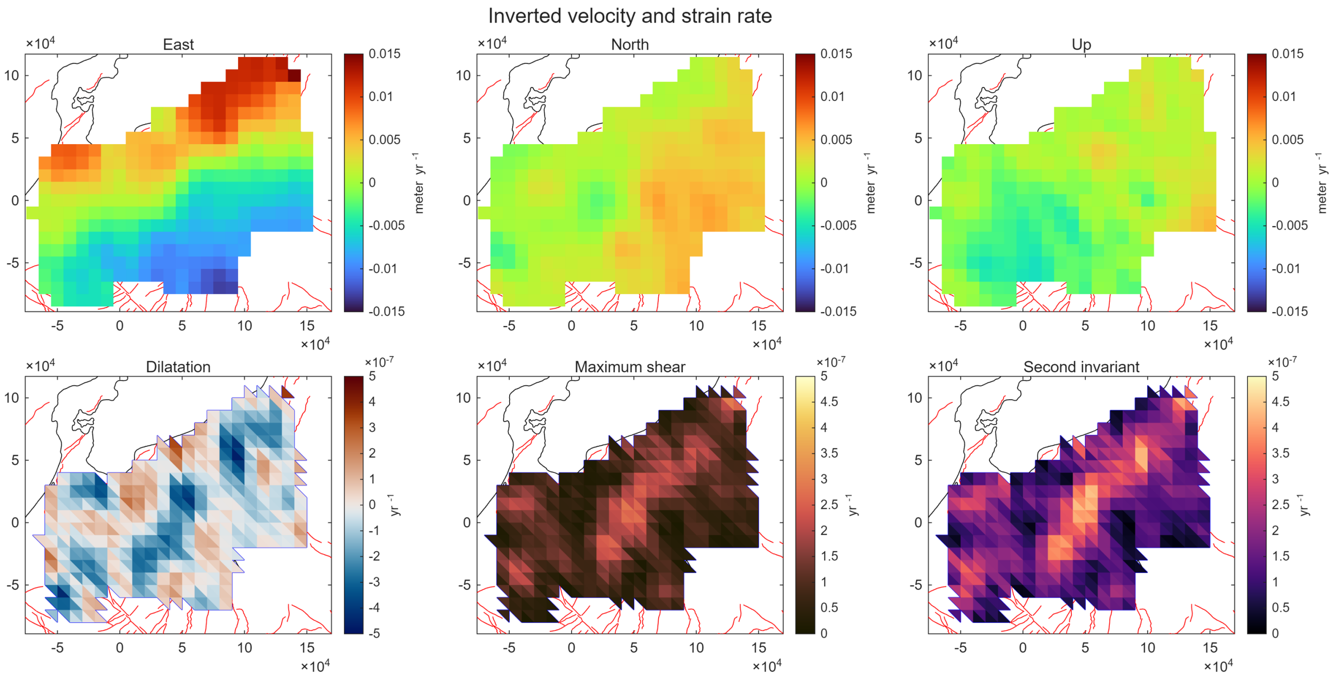

Figure S9. Three-dimensional velocity and strain rate fields derived from the simultaneous inversion of GNSS and InSAR line-of-sight velocities[14,15]. The triangular mesh is defined on a regular grid with ~ 10 km spacing. The smoothing factor κ is set to 10^8. Maps in this figure were generated using the MATLAB software R2025b (https://www.mathworks.com/products/matlab.html, accessed on 6 April 2026).

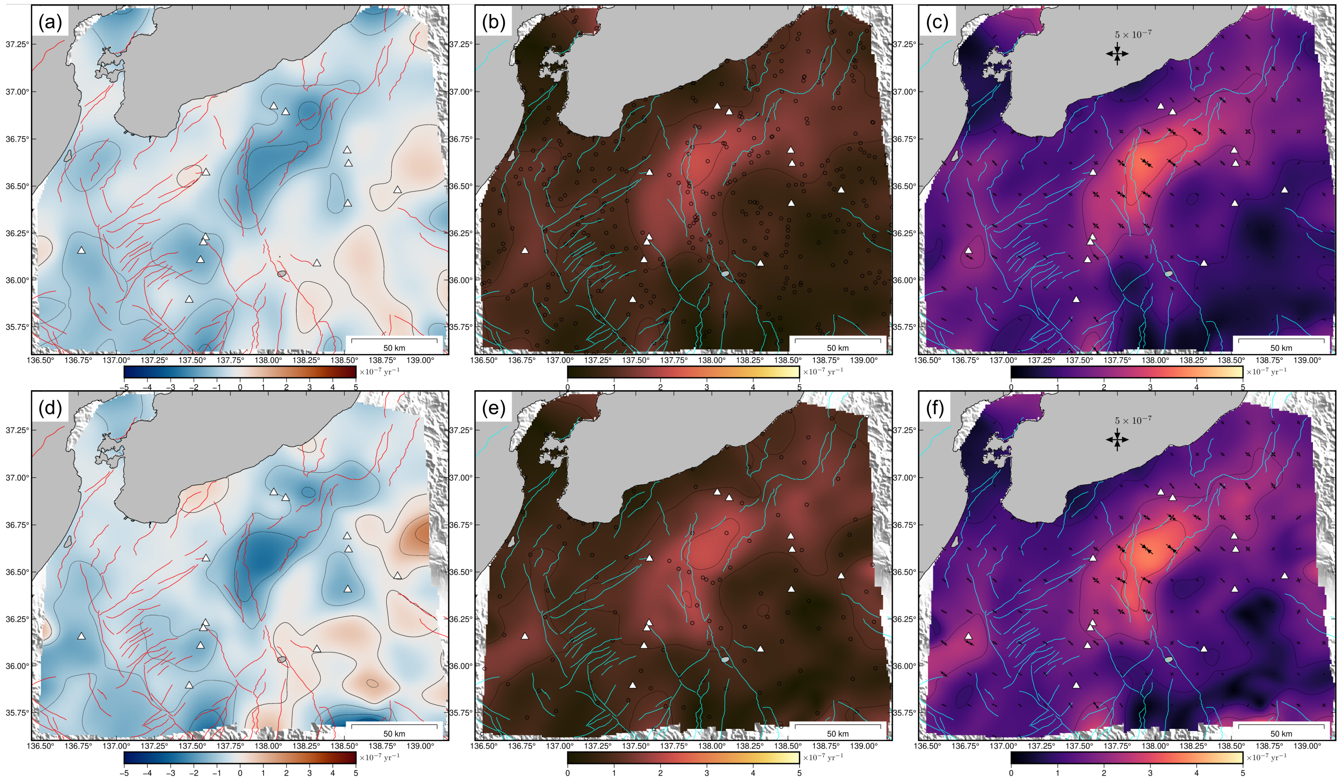

Figure S10. GNSS strain rates estimated by a conventional method[16,17]. The distance decay constant is set to 15 km. (**a–c**) Invariants of the strain tensor (dilatation, maximum shear, and second invariant), calculated from the all GNSS networks (GEONET, Universities, SB). (**d–f**) Invariants of the strain rate tensor, calculated from the GEONET stations only. The black open circles in (**b**) and (**e**) show the location of the GNSS stations. The coupled black arrows in (**c**) and (**f**) denote magnitude and directions of the principal strain rates. The white triangles indicate Holocene volcanoes. Red and cyan lines indicate active fault traces. The contour interval is 1×10^{-7} yr^{-1}. Maps in this figure were generated using the Generic Mapping Tools version 6 (https://www.generic-mapping-tools.org/, accessed on 6 April 2026).

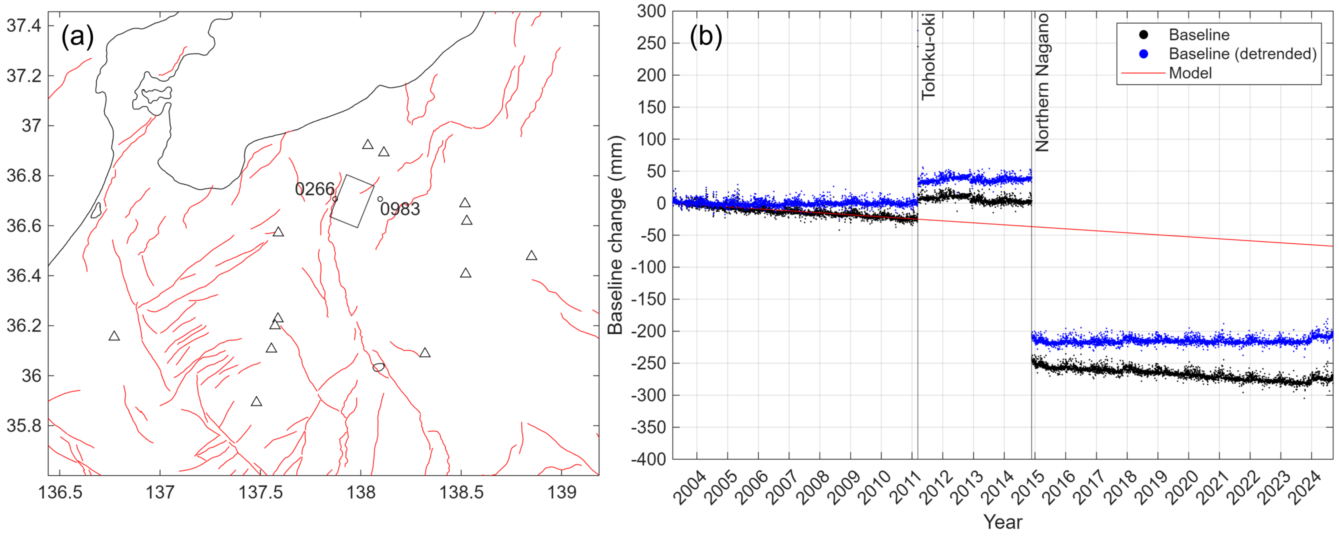

Figure S11. GNSS baseline length change. (**a**) Station locations. Inter-station distance is ~ 20 km. The black rectangle shows the location of the fault model of the 2014 Northern Nagano earthquake[13]. The white triangles mark Holocene volcanoes and the red lines indicate active fault traces. (**b**) Baseline length change. The black dots represent the baseline length time series. The red line denotes a linear trend estimated from the data before the 2011 Tohoku-Oki earthquake and the blue dots indicate detrended time series. The map was generated using the MATLAB software R2025b (https://www.mathworks.com/products/matlab.html, accessed on 6 April 2026).

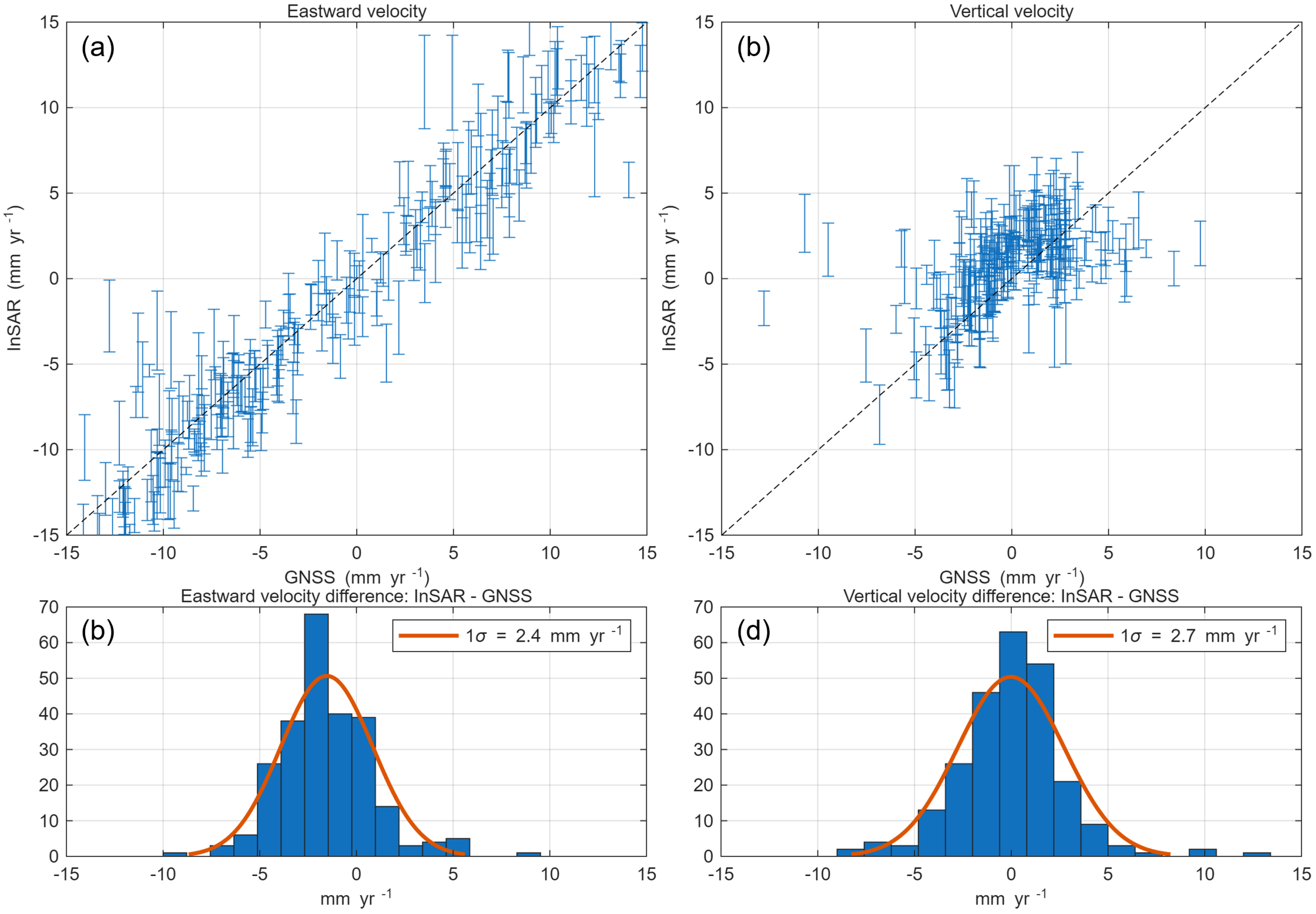

Figure S12. Comparison between InSAR- and GNSS-derived velocities. (**a, c**) Scatter plot of InSAR versus GNSS velocities. The black dashed line indicates the 1:1 correspondence between the two measurements. (**b, d**) Histogram of the velocity differences between InSAR and GNSS. The orange curve represents the best-fitting normal distribution.

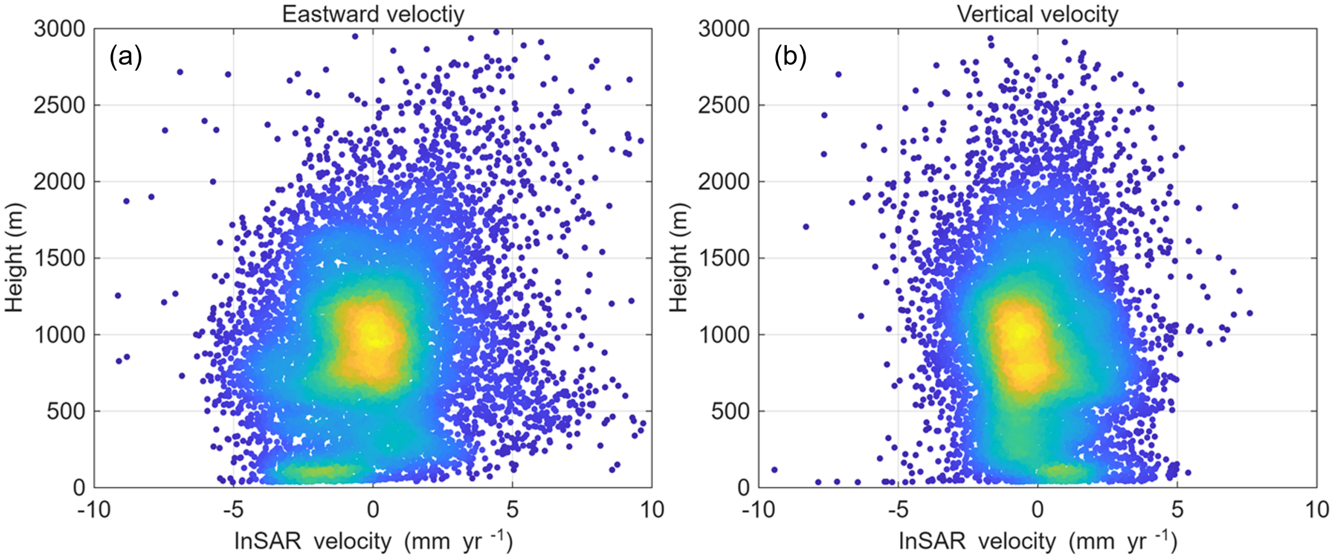

Figure S13. Height-dependence check of the average InSAR velocities. (**a**) Eastward velocity (Figure 3a), (**b**) vertical velocity (Figure 3b). Prior to plotting, long-wavelength velocity components were removed using a first-order polynomial fit. Marker colors indicate point density.

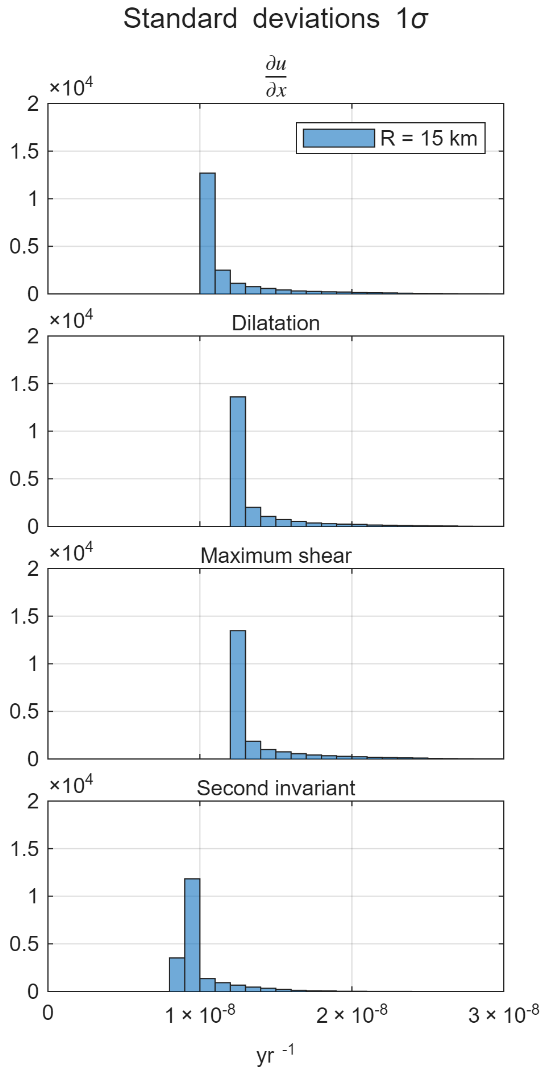

Figure S14. Histogram of the estimated standard deviations for the strain rate fields. The radius R is set to 15 km (Supplementary Texts S1, S3, and S4).

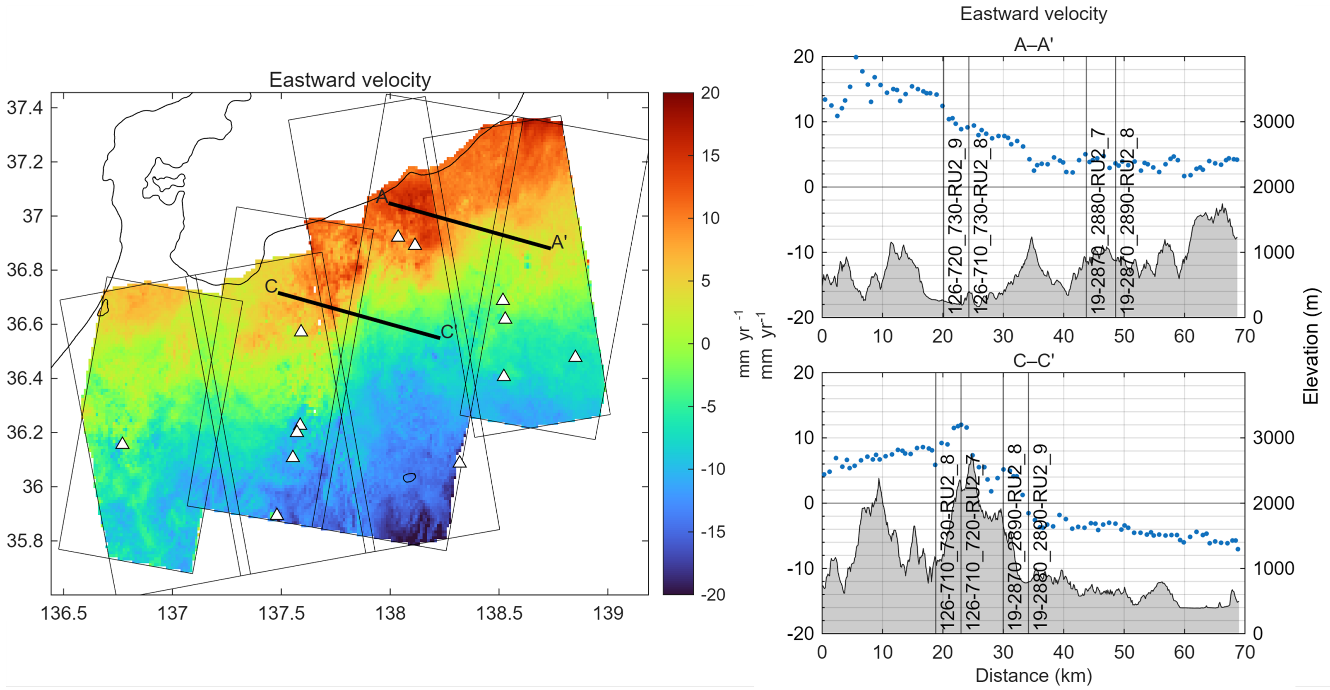

Figure S15. Left: InSAR eastward velocity. The black rectangle shows SAR acquisition frames. Right: InSAR eastward velocity along the profiles. The vertical lines indicate the edges of SAR frames. The black line with shaded area shows elevation in meters. The map was generated using the MATLAB software R2025b (https://www.mathworks.com/products/matlab.html, accessed on 6 April 2026).

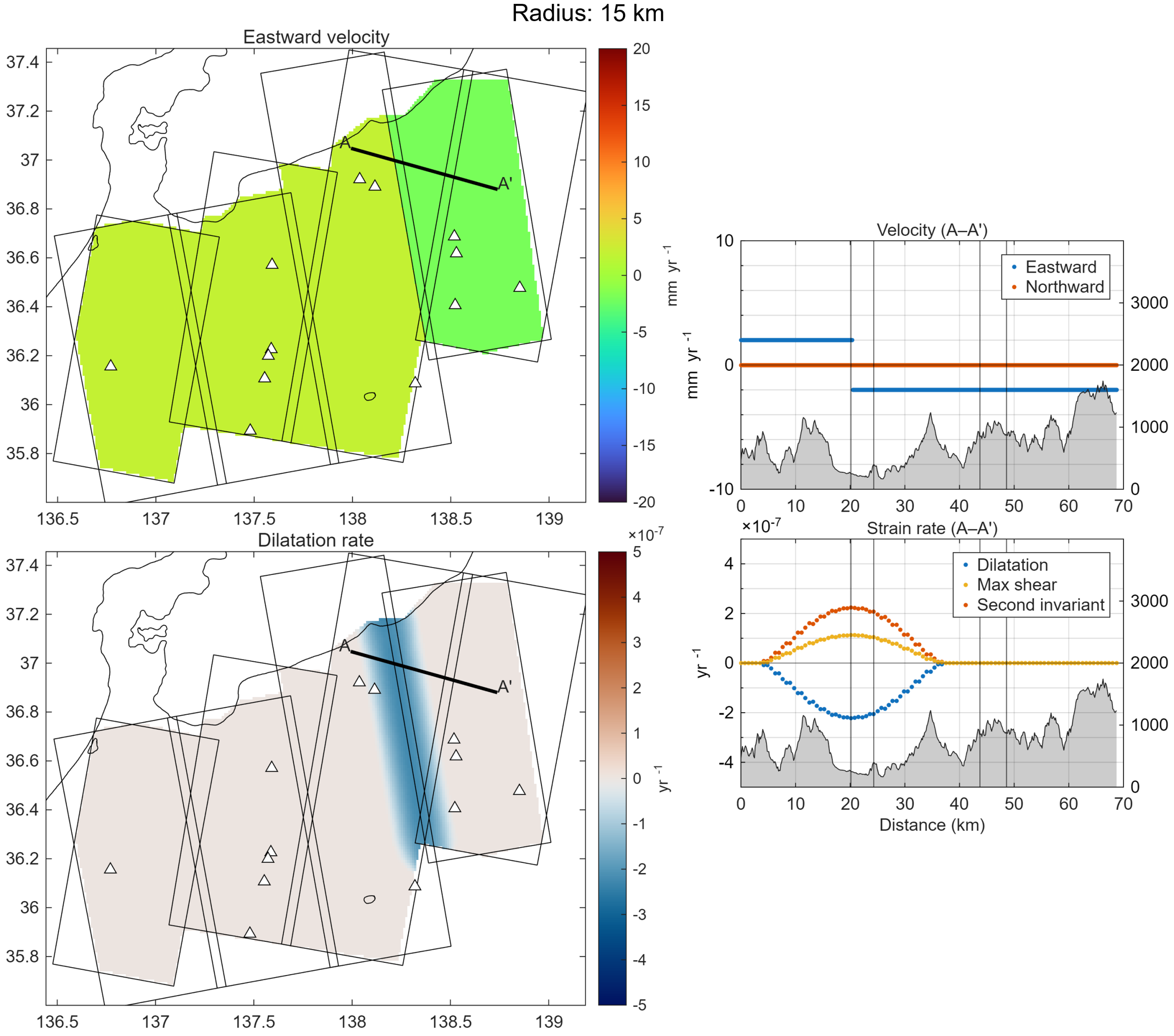

Figure S16. Effect of a swath gap in the strain rate estimates. Top left: an artificial velocity map (eastward), with a gap of 4 mm yr^{-1}. The black rectangles show SAR acquisition frames. Bottom left: corresponding strain rate (dilatation rate), calculated in the same method ($R = 15$ km). Top right: eastward and northward velocities along the profile. Bottom right: profile of the corresponding strain rates. The vertical lines indicate the location of the SAR frame edges. The black line with shaded area shows elevation. Maps in this figure were generated using the MATLAB software R2025b (https://www.mathworks.com/products/matlab.html, accessed on 6 April 2026).
